# Illumina MiSeq 16S amplicon sequence analysis of bovine respiratory disease associated bacteria in lung and mediastinal lymph node tissue

**DOI:** 10.1186/s12917-017-1035-2

**Published:** 2017-05-02

**Authors:** Dayle Johnston, Bernadette Earley, Paul Cormican, Gerard Murray, David Anthony Kenny, Sinead Mary Waters, Mark McGee, Alan Kieran Kelly, Matthew Sean McCabe

**Affiliations:** 1 Animal and Bioscience Research Department, Animal & Grassland Research and Innovation Centre, Teagasc Grange, Dunsany, Co. Meath, Ireland; 2 School of Agriculture Food Science and Veterinary Medicine, University College Dublin, Dublin, Belfield, Dublin 4 Ireland; 3 Department of Agriculture, Food and the Marine, Regional Veterinary Laboratory, Sligo, Co. Sligo Ireland; 4 Livestock Systems Research Department, Animal & Grassland Research and Innovation Centre, Teagasc Grange, Dunsany, Co. Meath, Ireland

**Keywords:** Bovine respiratory disease, lung microbiome, 16S sequencing, diagnostics

## Abstract

**Background:**

Bovine respiratory disease (BRD) is caused by growth of single or multiple species of pathogenic bacteria in lung tissue following stress and/or viral infection. Next generation sequencing of 16S ribosomal RNA gene PCR amplicons (NGS 16S amplicon analysis) is a powerful culture-independent open reference method that has recently been used to increase understanding of BRD-associated bacteria in the upper respiratory tract of BRD cattle. However, it has not yet been used to examine the microbiome of the bovine lower respiratory tract. The objective of this study was to use NGS 16S amplicon analysis to identify bacteria in post-mortem lung and lymph node tissue samples harvested from fatal BRD cases and clinically healthy animals. Cranial lobe and corresponding mediastinal lymph node post-mortem tissue samples were collected from calves diagnosed as BRD cases by veterinary laboratory pathologists and from clinically healthy calves. NGS 16S amplicon libraries, targeting the V3-V4 region of the bacterial 16S rRNA gene were prepared and sequenced on an Illumina MiSeq. Quantitative insights into microbial ecology (QIIME) was used to determine operational taxonomic units (OTUs) which corresponded to the 16S rRNA gene sequences.

**Results:**

*Leptotrichiaceae, Mycoplasma, Pasteurellaceae*, and *Fusobacterium* were the most abundant OTUs identified in the lungs and lymph nodes of the calves which died from BRD. *Leptotrichiaceae, Fusobacterium*, *Mycoplasma, Trueperella* and *Bacteroides* had greater relative abundances in post-mortem lung samples collected from fatal cases of BRD in dairy calves, compared with clinically healthy calves without lung lesions. *Leptotrichiaceae, Mycoplasma* and *Pasteurellaceae* showed higher relative abundances in post-mortem lymph node samples collected from fatal cases of BRD in dairy calves, compared with clinically healthy calves without lung lesions. Two *Leptotrichiaceae* sequence contigs were subsequently assembled from bacterial DNA-enriched shotgun sequences.

**Conclusions:**

The microbiomes of the cranial lung lobe and mediastinal lymph node from calves which died from BRD and from clinically healthy H-F calves have been characterised. Contigs corresponding to the abundant *Leptotrichiaceae* OTU were sequenced and found not to be identical to any known bacterial genus. This suggests that we have identified a novel bacterial species associated with BRD.

**Electronic supplementary material:**

The online version of this article (doi:10.1186/s12917-017-1035-2) contains supplementary material, which is available to authorized users.

## Background

Bovine respiratory disease (BRD) is associated with significant morbidity and mortality in cattle worldwide, and contributes to substantial economic losses [1–3]. Clinical signs associated with BRD typically include elevated rectal temperature, increased respiratory rate, nasal and ocular discharges, cough, dyspnea, decreased appetite and depression [2, 4, 5]. In Ireland, BRD is a leading cause of mortality in calves between one and 5 months of age [6].

BRD results from a multifactorial aetiology of infectious agents, host factors, environmental stress factors and their interactions [7–9]. Primary pathogens including bovine herpesvirus 1 (Bo-HV1), bovine respiratory syncytial virus (BRSV), bovine parainfluenza 3 virus (BPIV-3), bovine viral diarrhoea virus (BVDV), bovine coronavirus (BCoV) and *Mycoplasma bovis,* are thought to be generally responsible for the onset of BRD [1, 4, 10–12] although recent viral metagenomic NGS studies suggest that there are many more uncharacterised BRD-associated viruses than was previously thought [13, 14]. BRD-associated viral and *Mycoplasmal* pathogens damage the lungs by causing immunosuppression, ciliary dysfunction and cellular necrosis [15, 16]. This damage allows proliferation and colonisation by secondary bacterial pathogens, commonly including *Mannheimia haemolytica, Pasteurella multocida*, *Histophilus somni, Mycoplasma bovis* and *Trueperella pyogenes,* which are normally commensal in the nasopharyngeal region of cattle [1, 7, 11, 17–19]. These bacteria evade the lung’s remaining immune defences and their virulence factors cause progression of the disease if left untreated [7, 11, 15, 16].

Currently available vaccinations and antibiotic treatments are targeted against the known viruses and bacteria associated with BRD. Poor efficacy of vaccination and antimicrobial treatments against BRD associated bacteria [20–22] may partially be due to the involvement of unknown bacterial pathogens in the disease.

The methods that are currently used for identification of specific bacteria associated with fatal cases of BRD are culture, immunohistochemistry, in-situ hybridisation and multiplex and uniplex polymerase chain reaction (PCR) [23]. However, these techniques are time consuming and they have several limitations. Although culture is considered the gold standard for pathogen identification [24], the main limitation of this technique is that many bacterial species associated with BRD are difficult to culture [25, 26]. PCR has been demonstrated to be a more sensitive method than culture for identification of bacteria in bovine pneumonic lung tissue [27, 28]. Although real-time qPCR is commonly used in veterinary diagnostic laboratories, only known bacteria can be identified using this “closed reference” technique and each qPCR assay is generally designed to detect one individual bacterial species.

Next generation sequencing of 16S ribosomal RNA gene PCR amplicons (NGS 16S amplicon analysis) has been used for identification of bacteria present in diverse sample types including clinical isolates [29, 30], the bovine rumen [31], human nasal lavage [32] and nasopharyngeal swabs from feedlot cattle [26, 33]. As primers bind to conserved regions of the 16S ribosomal RNA (rRNA) gene [34] and phylogenetically variable regions are amplified and subsequently sequenced (typically generating 10,000–100,000 sequences per sample), identification of the bacteria present (both known and unknown) in the sample is possible at unprecedented depth [35].

The aim of this study was to use NGS 16S amplicon analysis to identify unculturable and previously unknown bacteria which may play a pathogenic role in BRD. For this we analysed DNA from post-mortem cranial lung lobe and mediastinal lymph node tissue from beef and dairy calves with BRD diagnosed as the cause of death, and from clinically healthy Holstein-Friesian (H-F) calves. We found highly abundant 16S sequence for which the closest match was *Leptotrichiaceae*. This putative *Leptotrichiaceae* 16S sequence was present in lung and lymph node tissue from fatal BRD cases and in lung lesions from clinically healthy calves but was absent in lesion-free lungs from clinically healthy calves. To our knowledge this is the first report of detection of this putative *Leptotrichiaceae* species in bovine lung lesions in cattle with BRD and also the first report of NGS 16S amplicon analysis of lung tissue and lung-associated lymph nodes in cattle with BRD.

## Methods

### Post-mortem tissue sample collection

Thirty-eight cranial lung lobe tissue samples were collected post-mortem from 32 beef calves and 6 dairy calves which were submitted from farms in three different regions in Ireland. Mediastinal lymph node tissue was also collected from 32 of these animals. BRD was diagnosed on gross examination at three regional veterinary laboratories (RVLs) (Athlone, Kilkenny and Sligo) by experienced pathologists. Bacteriological culture, virological and/or bacteriological PCR and histology for identification of aetiological agents (Table 1 and Additional file 1) were performed at the RVLs. In all cases, lung tissue samples were harvested from lesions (lesions were defined as macroscopic consolidation or abscessation of lung tissue) present on the cranial lobes of the lung. Approximately 1 g of each of these tissues was collected from each calf (comprising 13 beef and dairy breeds) (Table 1). Six of these post-mortem lung tissue samples were frozen at -80 °C immediately following collection. The remaining lung and lymph node post-mortem tissue samples were cut into slices less than 0.5 cm thick, placed in RNALater RNA Stabilization Reagent (Qiagen, Manchester, UK), stored at 4 °C overnight, and subsequently stored at -20 °C, according to the manufacturer’s instructions (Table 1).

**Table 1:** Description of post-mortem tissue samples collected at RVLs

Calf ID	Tissue types collected	Preservation method	Age (months)	Sex	Breed	County of farm of origin	RVL location	Lesions observed	Histology	RVL diagnosis
1	lung	frozen	6	F	SIX	Donegal	Sligo	✓	✓	Pneumonia (BRSV suspected)
2	lung	frozen	1	M	FRX	Donegal	Sligo	✓	x	Pneumonia and enteritis
3	lung	frozen	12	M	PTX	Donegal	Sligo	✓	✓	Pneumonia - IBR and *M. bovis*
4	lung	frozen	12	M	BBX	Donegal	Sligo	✓	✓	Pneumonia - IBR and *M. bovis*
5	lung	frozen	7	M	CHX	Donegal	Sligo	✓	✓	Pneumonia - *H. somni*
6	lung	frozen	6	F	CHX	Leitrim	Sligo	✓	x	Pneumonia - *M. haemolytica* and BPIV-3 virus
7	lung and lymph node	frozen	1	F	LM	Sligo	Sligo	✓	x	Pneumonia and navel infection - *M. bovis*, *Pseudomonas* spp.
8	lung and lymph node	RNALater	8	M	FR	Leitrim	Sligo	✓	x	Pneumonia - *T. pyrogenes*
9	lung and lymph node	RNALater	0	M	RBX	Donegal	Sligo	✓	x	Pneumonia - IBR, *H. somni,* *T. pyrogenes*
10	lung and lymph node	RNALater	3.5	M	FR	Leitrim	Sligo	✓	x	Pneumonia- *P. multocida* and*S. dublin*
11	lung and lymph node	RNALater	2.5	F	LM	Sligo	Sligo	✓	x	Pneumonia
12	lung and lymph node	RNALater	0	F	CHX	Carlow	Kilkenny	✓	x	Pneumonia
13	lung and lymph node	RNALater	3	F	AAX	Galway	Athlone	✓	✓	Pneumonia - *M. haemolytica*
14	lung and lymph node	RNALater	2	F	LMX	Westmeath	Athlone	✓	✓	Pneumonia and bacteraemia-*P. multocida*
15	lung and lymph node	RNALater	0	F	AAX	Offaly	Athlone	✓	✓	Pneumonia - IBR and necroticlaryngitis
16	lung and lymph node	RNALater	0	F	AAX	Offaly	Athlone	✓	✓	Pneumonia and pleurisy -*Pasteurella* spp., IBR and BRSV
17	lung and lymph node	RNALater	0	M	CHX	Longford	Athlone	✓	✓	Pneumonia and rotavirusenteritis
18	lung and lymph node	RNALater	1	M	CHX	Roscommon	Athlone	✓	✓	Pneumonia and navel ill -*T. pyogenes* (suspected virus and *Pasteurella*)
19	lung and lymph node	RNALater	0	M	WAX	Waterford	Kilkenny	✓	x	Pneumonia and rotavirus enteritis
20	lung and lymph node	RNALater	1.5	F	FR	Tipperary	Kilkenny	✓	✓	Pneumonia - *H. somni* and*M. bovis*
21	lung and lymph node	RNALater	3	M	LM	Kilkenny	Kilkenny	✓	✓	Pneumonia - *H. somni* and*M. bovis*
22	lung and lymph node	RNALater	0	F	CHX	Kilkenny	Kilkenny	✓	✓	Pneumonia - *P. multocida* /*H. somni* bronchopneumonia
23	lung and lymph node	RNALater	0	M	LMX	Laois	Kilkenny	✓	✓	Pneumonia - *H. somni* bronchopneumonia
24	lung and lymph node	RNALater	3	M	LMX	Sligo	Sligo	✓	x	Pneumonia
25	lung and lymph node	RNALater	2	M	CH	Mayo	Sligo	✓	x	Pneumonia - *Proteus* spp.
26	lung and lymph node	RNALater	3	F	CHX	Leitrim	Sligo	✓	x	Pneumonia - Endocarditis, enzootic pneumonia, inhalation pneumonia
27	lung and lymph node	RNALater	1.5	F	LMX	Sligo	Sligo	✓	x	Pneumonia - IBR
28	lung and lymph node	RNALater	1.5	F	AAX	Sligo	Sligo	✓	x	Pneumonia - IBR and *Proteus* spp.
29	lung and lymph node	RNALater	12	M	CHX	Donegal	Sligo	✓	✓	Pneumonia - (possible IBR) and meningitis
30	lung and lymph node	RNALater	1	M	AA	Sligo	Sligo	✓	x	Pneumonia - *M. bovis*
31	lung and lymph node	RNALater	2.5	F	LMX	Donegal	Sligo	✓	x	Pneumonia - *M. bovis*
32	lung and lymph node	RNALater	3	F	LMX	Kilkenny	Kilkenny	✓	✓	Pneumonia - interstitial pneumonia
33	lung and lymph node	RNALater	2.5	M	FR	Laois	Kilkenny	✓	x	Pneumonia - *P. multocida*
34	lung and lymph node	RNALater	5	M	AAX	Kilkenny	Kilkenny	✓	✓	Pneumonia - *P. multocida,* *T. pyrogenes* chronic bronchopneumonia
35	lung and lymph node	RNALater	4.5	F	LMX	Sligo	Sligo	✓	✓	Pneumonia - Pasteurellosis
36	lung and lymph node	RNALater	1	M	CHX	Longford	Athlone	✓	✓	Pneumonia - suppurative bronchial pneumonia.
37	lung and lymph node	RNALater	1.5	M	CHX	Galway	Athlone	✓	✓	Pneumonia - *M. haemolytica*
38	lung and lymph node	RNALater	3.5	F	AAX	Offaly	Athlone	✓	✓	Pneumonia - *H. somni,* *M. bovis, M. haemolytica,* *P. multocida, T. pyogenes*

Lesions were defined as macroscopic consolidation or abscessation of lung tissue.

*M* male, *F* female, *AA* Aberdeen Angus, *AAX* Aberdeen Angus cross, *BBX* Belgium Blue cross, *CH* Charolais, *CHX* Charolais cross, *FR* Friesian, *FRX* Friesian cross, *LM* Limousin, *LMX* Limousin cross, *PTX* Parthenaise cross, *RBX* Rotbunt cross, *SIX* simmental cross, *WAX* wagyu cross, ✓ = performed and confirmed bovine respiratory disease, x = not performed, *H. somni = Histophilus somni, M. bovis = Mycoplasma bovis, M. haemolytica = Mannheimia haemolytica, P. multocida = Pasteurella multocida, S. dublin = Salmonella dublin, T. pyrogenes = Trueperella pyrogenes,* BRSV = bovine respiratory syncytial virus, IBR = infectious bovine rhinotracheitis, BPIV-3 = bovine parainfluenza 3 virus.

### Bacteriology, virology and histology performed at RVLs

Aerobic bacteriology of swab samples from pneumonic lungs was performed on blood agar and McConkey agar at 37 °C for 2 days (*n* = 36) (Additional file 1), as described by Murray et al. [36]. Culture on chocolate agar under 8% CO_2_ for 3 days and on xylose lysine desoxycholate agar (following selenite enrichment) for 2 days at 37 °C was also conducted for the identification of *Histophilus somni* (*n* = 13) and *Salmonella* species (*n* = 24), respectively (Additional file 1) [36].

Lung and tracheal mucosa were submitted for detection of *Mycoplasma bovis* (*n* = 15), *Histophilus somni* (*n* = 11), *Mannheimia haemolytica* (*n* = 4) and *Pasteurella multocida* (*n* = 1), by real-time qPCR analyses (Additional file 1), which were performed as previously described [28, 37–39]. Real-time qPCR was also used to identify Bo-HV1 (*n* = 36) and reverse transcriptase qPCR was used to identify BRSV (*n* = 38), BPIV-3 (*n* = 35) and BCoV (*n* = 34) (Additional file 1) as previously described [12]. Positive and negative (no template) controls were included in each qPCR assay.

Sections for histology were taken from the border of grossly visible lesions on the cranial lobe (*n* = 21) (Table 1 and Additional file 1). Sections were fixed by placing them in 10% neutral-buffered formalin for 4 days, embedding them in paraffin wax, cutting them with a microtome and staining them with hematoxylin and eosin, as previously described by Murray et al. [36].

### Clinically healthy calves: health assessments

To serve as a comparison, 20 lung (cranial lobe) and 20 corresponding mediastinal lymph node tissue samples were also sourced from clinically healthy H-F calves which were slaughtered at approximately 2.5 months of age. These calves all recorded good performance prior to slaughter and achieved an average daily weight gain within a 0.25 standard deviation of their expected average daily gains.

Clinical health assessments of these calves were performed on the morning before slaughter using the Wisconsin calf health scoring criteria (https://www.vetmed.wisc.edu/dms/fapm/fapmtools/8calf/calf_respiratory_scoring_chart.pdf) to determine a respiratory score. A calf was considered to have respiratory disease if it had a respiratory score greater than or equal to 5 as it was then showing at least two signs of respiratory disease [5]. A full clinical history was available for these calves including a record of veterinary treatments for BRD.

### Clinically healthy control calves: collection of post-mortem tissue samples

The lungs were harvested from the calves immediately following slaughter at Teagasc Ashtown Research Centre. All surfaces and forceps were cleaned with 20% Domestos extended germ kill bleach (1% final sodium hypochlorite solution) (Unilever, Surrey, UK Ltd) Virusolve® + (Amity International Healthcare; Barnsley, UK), 70% ethanol and RNaseZAP™ (Sigma-Aldrich® Ireland Ltd., Wicklow, Ireland) initially and between samples.

The lungs were visually examined and the presence and number of lesions was recorded. Tissue samples from the cranial lobe and mediastinal lymph node were removed using sterile scalpel blades and forceps. Tissue samples were cut into slices less than 0.5 cm thick, placed in RNALater RNA Stabilization Reagent, stored at 4 °C overnight, and subsequently stored at -20 °C, according to the manufacturer’s instructions. When lesions were present on the cranial lobe, the lesions, rather than the lesion-free regions, were sampled.

### DNA extraction

DNA extraction from post-mortem tissue samples was carried out in a class II biosafety cabinet. Forceps, homogeniser and all work surfaces were cleaned initially and between samples using 20% sodium hypochlorite solution, Virusolve®+, 70% ethanol and RNaseZAP™. Homogeniser blades were sonicated for 10 min at 60 °C in molecular grade water (Sigma, Ireland), then rinsed by running while submerged in 75% ethanol and molecular grade water. DNA was extracted from post-mortem tissue samples using the Qiagen QIAamp Cador pathogen mini kit (with pre-treatments T2 (enzymatic digestion of tissue) and B1 (for difficult-to-lyse bacteria in pre-treated tissue)) (Qiagen, Manchester, UK) according to the manufacturer’s instructions with some modifications to pre-treatment T2.

A small piece of each tissue sample was removed using sterile scalpel blades and forceps, placed in a sterile Petri-dish, weighed, immediately submerged in buffer ATL (Qiagen, Manchester, UK) (360 μl per 50 mg tissue) and homogenised using a hand-held homogeniser (PRO 200, Bio-Gen Series; PRO Scientific Inc. Oxford, USA). Following homogenisation, 205 μl homogenised tissue was transferred to a 1.5 ml microcentrifuge tube (sterile) and 20 μl proteinase K was added. The microcentrifuge tube was placed in a shaking incubator overnight (300 rpm, 56 °C).

Pre-treatment B1 and the subsequent purification of pathogen nucleic acids from fluid samples were carried out according to the manufacturer’s instructions. Following nucleic acid extraction, RNA was removed using RNaseA solution, 4 mg/ml (Promega, Southampton, UK). This was achieved by adding 5 μl RNaseA solution to 100 μl of the sample’s purified nucleic acid eluate and incubating at 37 °C for 20 min. Subsequently, the DNA was purified using a Zymo genomic DNA clean & concentrator™-10 kit (Zymo Research Corp, Irvine, CA, USA), according to the manufacturer’s instructions. A Nanodrop spectrophotometer (NanoDrop Technologies, Wilmington, DE, USA) was used to quantify the DNA. All waste was autoclaved appropriately prior to disposal.

### 16S amplicon library preparation and sequencing

One hundred and sixteen 16S rRNA gene amplicon libraries (including 6 water control libraries) were prepared by PCR amplification of an approximate 467 bp region within the hypervariable (V3-V4) region of the 16S rRNA gene in bacteria, from 50 ng of each of the extracted and purified DNA from lung and lymph node tissue, and molecular grade water (non-template control), respectively, according to the Illumina 16S metagenomic sequencing library protocol, with modifications.

PCR was initially performed with broad spectrum 16S rRNA primers (forward primer: 5′-TCGTCGGCAGCGTCAGATGTGTATAAGAGACAG*CCTACGGGNGGCWGCAG*-3′, reverse primer: 5′-GTCTCGTGGGCTCGGAGATGTGTATAAGAGACAGGACTACHVGGGTATCTAATCC-3′) [34], using Kapa HiFi HotStart 2× ReadyMix DNA polymerase (Kapa Biosystems Ltd., London, UK). Cycle conditions were 95 °C (3 min), then 35 cycles of 95 °C (30 s), 63 °C (30 s), 72 °C (30 s), then a final extension of 72 °C (5 min). Libraries were purified using AMPure XP beads (LABPLAN; Naas, Ireland) according to the Illumina 16S metagenomic sequencing library protocol. Dual indices and Illumina sequencing adapters from the Illumina Nextera XT index kits v2 B and C (Illumina, San Diego, USA) were added to the target amplicons in a second PCR step using Kapa HotStart HiFi 2× ReadyMix DNA polymerase (Kapa Biosystems Ltd., London, UK). Cycle conditions were 95 °C (3 min), then 9 cycles of 95 °C (30 s), 55 °C (30 s), 72 °C (30 s), then a final extension of 72 °C (5 min). Libraries were again purified using AMPure XP beads (LABPLAN; Naas, Ireland) according to the Illumina 16S metagenomic sequencing library protocol.

Libraries were measured for purity and quantity on a Nanodrop 1000 spectrophotometer. The barcoded amplicon libraries were combined in equal concentrations into a single pool according to their Nanodrop quantification measurement. Two μl of each negative control library was added to the pool. The library pool was then quantified using the KAPA SYBR FAST Universal qPCR kit with Illumina Primer Premix (Kapa Biosystems Ltd., London, UK) and the size was assessed with an Agilent DNA 1000 Kit (Agilent Technologies Ireland Ltd., Dublin, Ireland) on an Agilent 2100 Bioanalyser (Agilent Technologies Ireland Ltd., Dublin, Ireland).

The library pool was diluted and denatured according to the Illumina MiSeq library preparation guide. The amplicon library (8 pM) was spiked with 30% denatured and diluted PhiX Illumina control library version 3 (12.5 pM). The sequencing run was conducted on the Illumina MiSeq using the 500 cycle MiSeq reagent kit (version 2) with paired 250 bp reads. All sequence data produced in this study has been deposited to NCBI SRA repository and are available through series accession number SRP080306.

### Bioinformatic analysis of amplicon library sequences

Raw sequence reads for all samples in the study were quality controlled using the BBduk (https://sourceforge.net/projects/bbmap/) Java package. This was used to trim low quality bases (<20 Phred score) from the 3′ end of sequence read pairs and identify and remove adaptor sequences. Illumina paired reads with an insert size (length of template molecule) that was shorter than the sum of the lengths of read 1 and read 2 were merged into a single, longer read. Size selection of 467 bp ±20 bp sequences was performed with an in-house Perl script.

The wrapper package Quantitative Insights Into Microbial Ecology (QIIME) [40] was used to determine the operational taxonomic units (OTU)s which corresponded to the 16S rRNA gene sequences in each sample. Sequences were clustered into individual OTUs at a default similarity level of 97% using an open reference picking strategy, and subsequently, a single representative sequence from each clustered OTU was used to align to the Greengenes database. The RDP Classifier [41], using a minimum confidence cut off of 0.8, was used to determine the taxonomic classification for each OTU. Any OTUs with fewer than 50 sequences across all samples were excluded from further analysis. All OTUs with only a single read count in any sample were removed from the analysis.

### Statistical analysis

Differences between relative abundances and the presence of bacterial OTUs between post-mortem lung tissue frozen at -80 °C upon collection and post-mortem lung tissue preserved with RNA-Later, from beef calves which died from BRD, were calculated in GraphPad Prism 6 (version 6.04) using the Mann-Whitney U test and the Fisher’s exact test, respectively.

The Mann-Whitney U test and the Fisher’s exact test in GraphPad Prism 6 (version 6.04) were used to determine if differences existed between the relative abundances and the presence, respectively, of bacterial OTUs in the lung and lymph node tissues between dairy calves which died from BRD and clinically healthy dairy calves without lung lesions present at slaughter.

Stacked bar charts displaying relative abundances of bacterial OTUs in the specific tissue types were prepared in GraphPad Prism 6 (version 6.04).

### Microbial DNA enrichment and metagenomics shotgun sequencing

A lung sample from calf number 23 was chosen for shotgun sequencing as it had a high prevalence of a genus of interest (*Leptotrichiacea*) and did not contain many other different bacterial genera. DNA was suspended in Tris-EDTA buffer and re-quantified using the Nanodrop spectrophotometer. One μg of the DNA was enriched for microbial DNA by selective binding and removal of the CpG-methylated host DNA using the NEBNext® Microbiome DNA Enrichment Kit (New England Biolabs (UK) Ltd., Hitchin, Herts, UK). The microbial-enriched DNA sample was diluted and quantified with an Agilent high sensitivity DNA kit (Agilent Technologies Ireland Ltd., Dublin, Ireland) on an Agilent 2100 Bioanalyser.

### Shotgun library preparation and sequencing

One nanogram of microbial-enriched DNA was used to prepare a library using the Illumina Nextera®XT DNA Library Preparation Kit, according to the manufacturer’s instructions. Estimation of library size was performed using an Agilent high sensitivity DNA kit on an Agilent 2100 Bioanalyser and the library was quantified using the KAPA SYBR FAST Universal qPCR kit with Illumina Primer Premix. The library was diluted and denatured according to the Illumina MiSeq library preparation guide. The 8 pM shotgun library was spiked with 1% denatured and diluted PhiX Illumina control library version 3 (12.5 pM). A 250 bp paired end sequencing run was conducted on an Illumina MiSeq using a 500 cycle MiSeq reagent kit (version 2).

### Bioinformatic analysis of microbial DNA enriched shotgun sequence

The BBduk (https://sourceforge.net/projects/bbmap/) Java package was used to trim low quality bases (<20 Phred score) from the 3′ end of raw sequence read pairs and to identify and remove adaptor sequence. All surviving read pairs were mapped to the bovine genome (v.UMD3.1) using bwa [42] to identify host sequences. Approximately 100,000 paired-end sequences with no full-length alignment to the bovine genome were retained for *de novo* assembly. Contig assemblies were generated using OMEGA metagenome assembler (V1.0.2) with default settings. BLAST (BLASTN 2.3.0+) searches against the non-redundant nucleotide collection database on the NCBI website [43, 44] were conducted to determine known bacterial sequences that were most similar to the contig assemblies.

## Results

### Health assessments of clinically healthy calves

All of the clinically healthy H-F calves were assessed as clinically healthy on the day of slaughter and had respiratory scores based on the Wisconsin calf health scoring criteria [5] of less than 5 (Additional file 2).

### Lung lesions in clinically healthy calves

Lesions (macroscopic consolidation or abscessation of lung tissue) were observed on lungs from 12 H-F calves from the ‘clinically healthy’ group immediately post-slaughter (Additional file 3). Seven of these calves had lesions only on the cranial lobes, two had lesions on the middle lobe, two had lesions on both cranial and middle lobes and one calf had lesions on both the cranial and caudal lobes (Additional file 3). Eight calves from the ‘clinically healthy’ group had healthy lungs with no lesions present (Additional file 3).

In total, 5 out of the 20 H-F calves from the ‘clinically healthy’ group had recorded incidents of BRD (Additional file 3). These calves were all treated with Nuflor (Merck Animal Health, New Jersey) and Metacam (Boehringer Ingelheim Vetmedica GmbHm, Germany) and one calf was also treated with Dexamethasone (Norbrook, UK) and Zuprevo (Merck Animal Health, New Jersey). These antibiotic treatments were received at least 41 days before slaughter. All calves with previous recorded incidents of BRD had lung lesions present at slaughter and 7 calves with no recorded incidents of BRD also had lung lesions observed at slaughter (Additional file 3). Furthermore, one calf with lung lesions received Primidoxine (Norbrook, UK) and ASGold (Volac, UK) for treatment of enteritis 70 days before slaughter and one calf with lung lesions present received Dexamethasone and Nuflor 10 days before slaughter due to a reaction to vaccination.

### Negative control amplicon library PCR

To test for contaminating 16S DNA in the PCR reagents that we used for generation of amplicon libraries, we conducted six negative (non-template) control amplicon library reactions by substituting template DNA for molecular grade water. Following PCR amplification and agarose gel electrophoresis, no amplicon bands were observed in the gel (Additional file 4 (A)) and no peaks were observed on the electropherogram images obtained from the DNA 1000 chip on an Agilent 2100 Bioanalyser (Additional file 4 (C)). In contrast, bands of the expected amplicon size (467 bp) were observed on the gel for the selected lung tissue DNA libraries and the pooled lung and lymph node tissue DNA libraries (Additional file 4 (A)). Furthermore, the electropherogram images for the selected lung tissue DNA libraries and the pooled lung and lymph node tissue DNA libraries also confirmed that these libraries contained DNA fragments corresponding to the expected amplicon size (Additional file 4 (B)).

### Taxonomic classification

One hundred and fifteen bacterial OTUs were identified overall. Seventy-two were identified to genus level. Additionally, 32 OTUs could only be identified as far as family level, 7 OTUs could only be identified to order, 2 to class level and 2 only to phylum level (Additional files 5 and 6).

### Low numbers of classifiable reads in clinically healthy animals

**Fig. 1:**
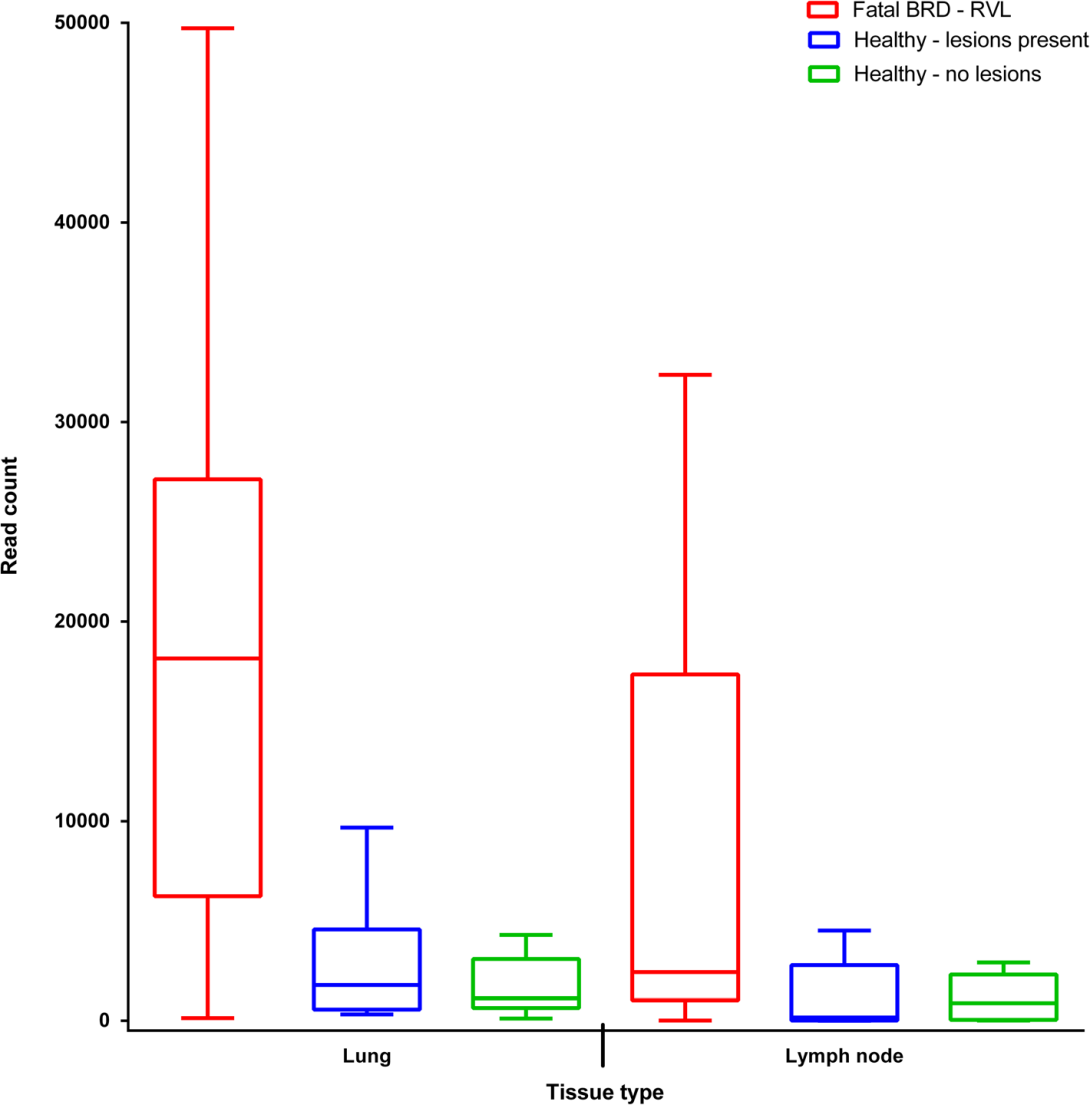
Number of reads (classified as bacterial) within post-mortem lung and mediastinal lymph node tissues corresponding to each sample type; calves which died from the BRD (*n* = 38), and healthy Holstein-Friesian calves (with (*n* = 12) and without (*n* = 8) lung lesions). Fatal BRD - RVL = samples from calves with the bovine respiratory disease complex collected at regional veterinary laboratories. Healthy – lesions present = samples from healthy Holstein-Friesian calves with lung lesions. Healthy – no lesions = samples from healthy Holstein-Friesian calves without lung lesions. Boxplot = Tukey boxplot (lowest datum (whisker) is within a 1.5 interquartile range of the lower quartile, highest datum (whisker) is within a 1.5 interquartile range of the upper quartile, outliers (individual points) fall above and below these whiskers)

Even though equimolar amounts of each library were added to the pool that was sequenced on the MiSeq, the number of reads that were assigned to bacterial OTUs that could be classified by QIIME were much higher in lung and lymph node tissue from fatal BRD cases than from clinically healthy animals (Fig. 1). Unexpectedly, the PCR amplicon library yields were not different between BRD cases and clinically healthy animals. This indicates that in the clinically healthy animals, the 16S primers were amplifying a sequence in the lung and lymph node that was not on the 16S Greengenes database.

### OTU abundance in lung tissue

The top 5 most abundant OTUs in the post-mortem lung samples from the beef calves which died from BRD were classified by QIIME as *Pasteurellaceae* ((mean relative abundance (S.E.M)) 22.4 (5.84)%), *Mycoplasma* (15.1 (5.03)%)*, Leptotrichiaceae* (14.9 (4.40)%), *Clostridium* (6.4 (4.04)%) and *Fusobacterium* (5.1 (2.14)%). The top 5 most abundant OTUs in the post-mortem lung tissue samples from dairy calves which died from BRD were classified as *Leptotrichiaceae* (26.0 (14.0)%), *Mycoplasma* (22.0 (11.00)%), *Pasteurellaceae* (16.0 (7.40)%)*, Fusobacterium* (15.0 (8.70)%) and *Bacteroides* (8.1 (6.00)%). The most abundant OTUs within the lung tissue samples from the clinically healthy H-F calves with lung lesions were classified as *Leptotrichiaceae* (20.0 (9.90)%), *Mycoplasma* (15.5 (7.42)%)*, Prevotella* (12.3 (4.31)%) *Pasteurellaceae* (10.2 (6.44)%)*,* and *Actinobacillus* (4.6 (4.59)%)*. Leptotrichiaceae* and *Fusobacterium* were undetectable in the lesion-free lung samples from the clinically healthy H-F calves. The most abundant OTUs in lesion-free samples were *Prevotella* (30.7 (7.56)%)*, Bacteroides* (7.4 (7.38)%), *Pasteurellaceae* (6.6 (5.03)%)*, S24–7* (6.0 (5.24)%), and *Clostridium* (5.5 (3.78)%)*.*

### OTU abundance in lymph node tissue

The most abundant OTUs in lymph node tissue from beef calves which died from BRD were classified as *Pasteurellaceae* (18.0 (5.66)%)*, Clostridium* (9.6 (5.30)%), *Fusobacterium* (7.3 (2.84)%), *Leptotrichiaceae* (6.8 (2.66)%) and *Prevotella* (6.0 (2.90)%). *Clostridium* (21.9 (19.11)%), *Fusobacterium* (21.7 (14.81)%), *Mycoplasma* (21.3 (19.67)%)*, Leptotrichiaceae* (13.7 (10.68)%) and *Pasteurellaceae* (8.1 (5.46)%) were the most abundant OTUs in post-mortem lymph node samples from dairy calves which died from BRD. The most abundant OTUs in post-mortem lymph node samples from the clinically healthy H-F calves with lung lesions were classified as *Prevotella* (21.9 (10.40)%), *Lysinibacillus* (16.7 (11.24)%), *Cupriavidus* (8.4 (8.10)%), *Clostridium* (7.5 (5.64)%) and *Leptotrichiaceae* (6.8 (3.79)%). Notably, *Leptotrichiaceae* and *Fusobacterium* OTUs were not detected in the lymph node samples from the clinically healthy H-F calves without lung lesions. *Prevotella* (42.2 (11.63)%)*, Enterobacteriaceae* (12.5 (12.50)%), *Bacteroides* (10.0 (9.96)%), *Succinivibrionaceae* (7.9 (4.73)%), and *Clostridiales* (3.9 (2.27)%) were the most abundant OTUs that were detected in these lymph nodes*.*

### Differences between lung and lymph node microbiomes

**Fig. 2:**
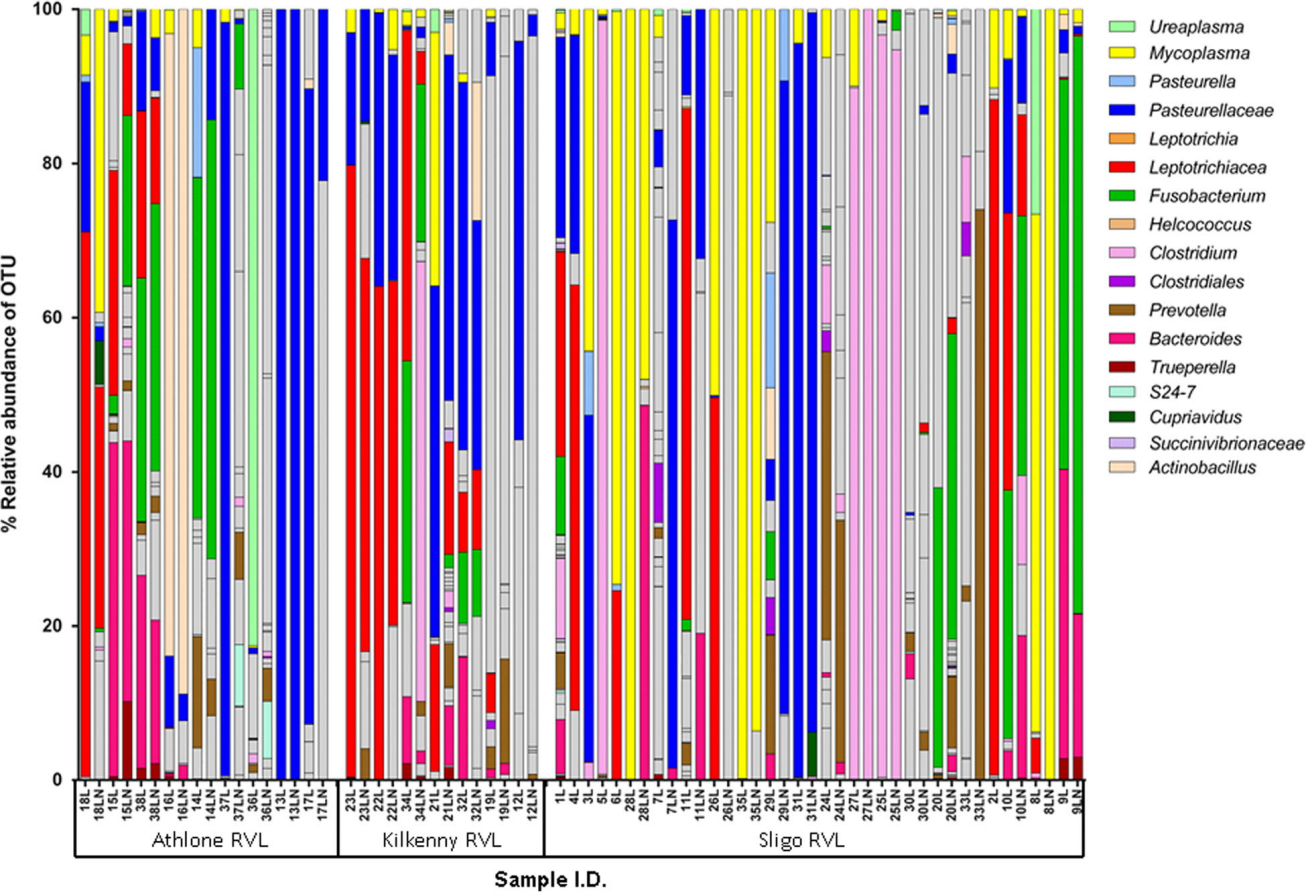
Percentage relative abundance of bacteria in post-mortem lung (L) and corresponding lymph node (LN) samples from calves which died from bovine respiratory disease. Adjacent profiles of L and LN are from the same animal. Athlone = sample collected at Athlone regional veterinary laboratory (RVL). Kilkenny = sample collected at Kilkenny RVL. Sligo = sample collected at Sligo RVL. Grey bars represent infrequently occurring OTUs (see Additional file 6 for full details of all bacterial OTU classifications)

Although OTU relative abundances in lung and lymph node were similar in some calves, the microbiomes of these two tissues were quite different in most of the animals. For example, more than half of the OTUs were identified as *Clostridium* in the lymph node of calf number 34 whereas no *Clostridium* was detected in the lung of this animal. Furthermore, *Ureaplasma* was the main genera detected in the lung of calf number 36 but this genera was not detected in the lymph node of this animal (Fig. 2).

### Undetected and detected genera in clinically healthy lesion-free lungs and lymph nodes

**Fig. 3:**
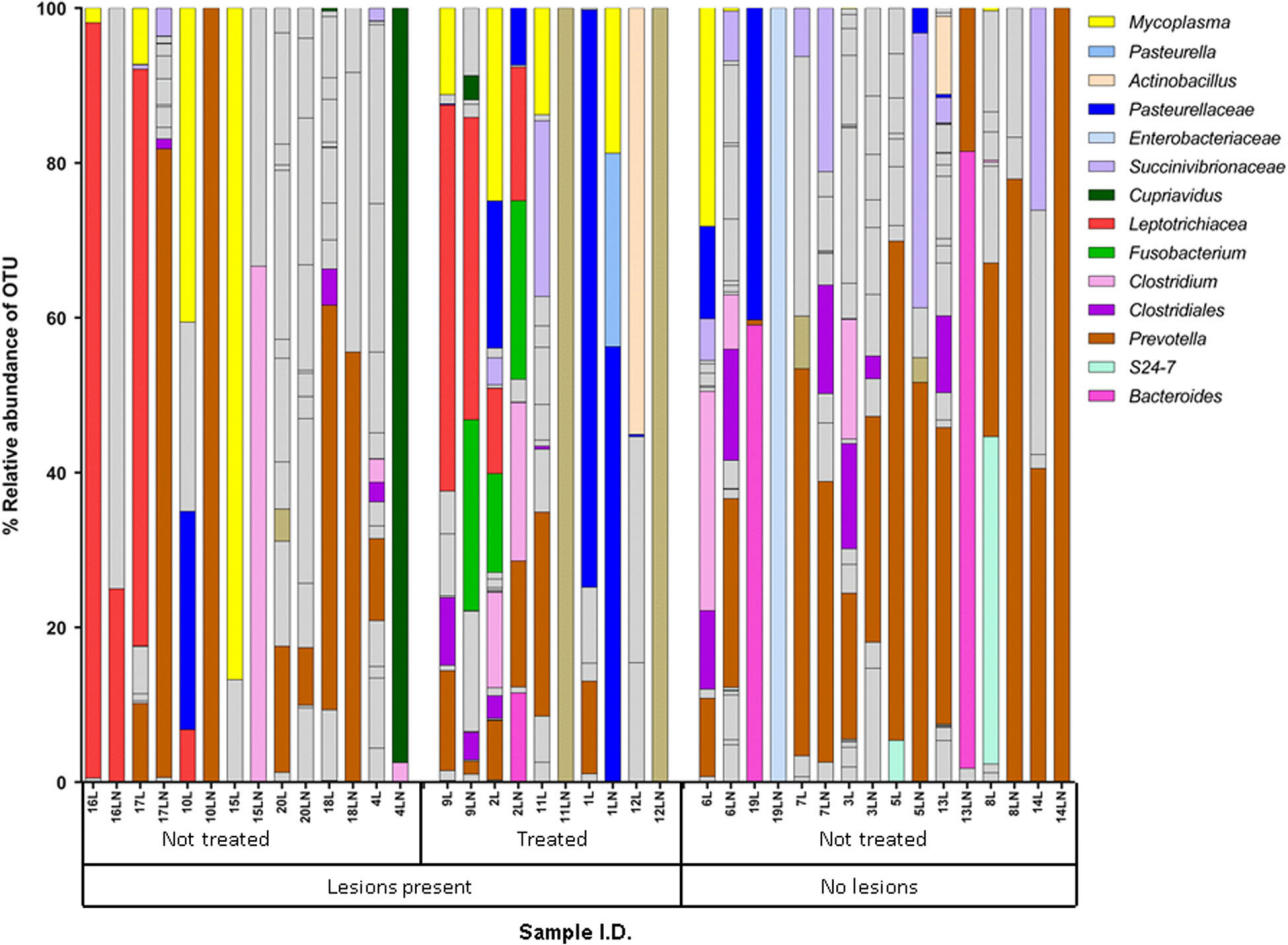
Percentage relative abundance of bacteria in post-mortem lung (L) and corresponding lymph node (LN) samples from clinically healthy H-F calves with lung lesions (*n* = 12) and without lung lesions (*n* = 8). Adjacent profiles of L and LN are from the same animal. The first seven calves received antibiotic treatment during their lifetimes. Grey bars represent infrequently occurring OTUs (see Additional file 6 for full details of all bacterial OTU classifications)

OTUs that were classified as *Leptotrichiaceae, Fusobacterium, Pasteurella, Trueperella, Helcococcus,* and *Ureaplasma* were abundant in lung and lymph node tissue samples from both beef and dairy calves that died from BRD, but these genera were not detected in either lung or lymph node tissue samples from the subset of clinically healthy H-F calves which had no observable lung lesions (*n* = 8) (Figs. 2 and 3).

At least two putative BRD-associated genera were detected in the lesion-free lungs. *Mycoplasma* was detected in three lung (6L, 19L and 13L) and one lymph node tissue sample (5LN) (Fig. 3)*.* Although relative abundances of *Mycoplasma* were reasonably high in 6 L and 19L, the read counts of this genera were negligable (6L = 308 reads) compared to the fatal BRD cases, several of which had read counts of >10,000 (Additional file 5). Sequences that were classified as *Pasteurellaceae,* a family containing species that are commonly associated with BRD were detected in three lung tissue and one lymph node tissue sample from the lesion-free calves (Fig. 3) but, again, compared to the fatal BRD cases these were negligable. The most abundant bacterial genus detected in the lesion-free samples was *Prevotella* which is more commonly associated with the rumen. However, the average raw read counts for *Prevotella* were not significantly different (*P* > 0.05) between the fatal BRD (average 212, standard deviation 446) cases and the lesion-free animals (average 498, standard deviation 610).

### Comparison of OTU abundance between lung samples preserved with RNALater and frozen lung samples

There were no differences in the relative abundances or the presence of bacterial OTUs within post-mortem lung tissue samples which were frozen at -80 °C immediately following collection and post-mortem lung tissue samples preserved with RNALater.

### Comparison of OTU abundance between dairy calves which died from BRD and clinically healthy calves

Bacterial OTUs associated with BRD including *Fusobacterium*, *Mycoplasma, Trueperella* and *Bacteroides,* and an OTU not yet associated with BRD (*Leptotrichiaceae*), had greater relative abundances and were present more often, among the lung tissue samples collected from the dairy calves which died from BRD compared with the lung tissue samples collected from the clinically healthy H-F calves with no lung lesions (*P* < 0.05) (Additional file 7).

*Leptotrichiaceae*, *Mycoplasma* and *Pasteurellaceae* had greater relative abundances (*P* < 0.05) (Additional file 8) and were present more often (*P* < 0.05) among the lymph node tissue samples collected from the dairy calves which died from BRD compared with the lymph node tissue samples collected from the clinically healthy H-F calves with no observable lung lesions.

### Comparison of 16S rRNA gene amplicon sequencing and RVL qPCR

This 16S rRNA gene amplicon sequencing assay identified many more bacteria present in post-mortem lung and lymph node tissue from calves which died from BRD than were identified from the same calves at the RVLs using both culture and uniplex PCRs on swab samples from the pneumonic lungs (Additional file 9). An OTU representing the species detected by PCR in RVLs was identified using the 16S rRNA gene amplicon sequencing assay in all but one case (calf I.D. = 30) (Additional file 9). However, in seven calves (calf I.D. = 9, 19, 22, 23, 32, 35, 37) the *Mycoplasma* OTU was identified using the 16S rRNA gene amplicon sequencing assay even though a PCR test at the RVLs had confirmed no *Mycoplasma bovis* present (Additional file 9).

The 16S rRNA gene amplicon sequencing assay identified bacterial OTUs in all ten calves (calf I.D. = 1, 5, 11, 12, 17, 30, 31, 32, 35, 36) which were culture negative at the RVLs (Additional file 9). Two cases of *Escherichia coli* (calf I.D. = 4, 27), one case of *Pseudomonas* species (calf I.D. = 7), one case of *Trueperella pyogenes* (calf I.D. = 18) and one case of *Salmonella dublin* (calf I.D. = 3) were cultured at RVLs but were not identified by the 16S rRNA gene amplicon sequencing assay (Additional file 9).

### Metagenomic shotgun sequencing of a microbial DNA enriched post-mortem lung tissue sample

One of the post-mortem lung DNA samples (from calf number 23) was enriched for microbial DNA and completely sequenced. However, despite microbial DNA enrichment, 99% of the reads in the run mapped to the bovine genome. Approximately 100,000 sequences did not map to the bovine genome and were assembled into contigs. Most of these contigs aligned to the PhiX174 bacteriophage genome but three contigs (contig 1 = 2647 bp, contig 2 = 1376 bp, contig 3 = 744 bp) aligned to bacterial sequences. PhiX sequence was from the pre-prepared non-indexed PhiX V3 library (supplied by Illumina) which was added, as a quality control, to the microbial DNA-enriched library prior to sequencing on the MiSeq. Fluorescence from the bases in the non-indexed PhiX clusters bleeds into the fluorescence in the adjacent indexed clusters. Additional file 10 contains the sequences of the contigs which aligned to bacterial sequences. These contigs were blasted against the non-redundant nucleotide collection database on the NCBI website using the BLASTN 2.3.0+ [45, 46] programme in order to determine the most similar known bacterial sequences.

The top 10 blast hits for each contig are shown in Table 2. No sequences on the database were found to be 100% identical to any of the complete assembled contigs (Table 2). The top hit for contig number 1 was *Sneathia amnii* strain SN35 (GenBank: CP011280.1) (Table 2). It was 92% identical to 99% of contig number 1 (Table 2). The top hit for contig number 2 was also *Sneathia amnii* strain SN35 (GenBank: CP011280.1) (Table 2). It was 93% identical to 99% of contig number 2 (Table 2). Interestingly, contig number 2 was 100% identical to two identical partial 16S rRNA gene sequences (GenBank: KR514404.1) obtained from uncultured bacterial clones in the bovine reproductive tract of farm animals at University College Dublin (Table 2). Contig number 3 was 99% identical to 100% of *Histophilus somni* strain 2336 23S ribosomal RNA gene, complete sequence (Table 2).

**Table 2:** Assembled bacterial contigs description and top BLAST hits

Contig number	Top 10 BLAST hits	Query cover	Percentage identity
1 (2647 bp)	1.*Sneathia amnii* strain SN35, complete genome CP011280.1	99%	92%
	2. *Streptobacillus moniliformis* DSM 12112, complete genome CP001779.1	96%	90%
	3. *Streptobacillus moniliformis* strain DSM 12112 23S ribosomal RNA gene, complete sequence NR_076688.1	96%	90%
	4. *Sebaldella termitidis* strain ATCC 33386 23S ribosomal RNA gene, complete sequence NR_076681.1	96%	89%
	5. *Sebaldella termitidis* ATCC 33386, complete genome CP001739.1	96%	89%
	6. *Leptotrichia buccalis* strain C-1013-b 23S ribosomal RNA gene, complete sequence NR_076664.1	96%	88%
	7. *Leptotrichia buccalis* DSM 1135, complete genome CP001685.1	96%	88%
	8. *Leptotrichia* sp. oral taxon 212 strain W10393, complete genome CP012410.1	96%	87%
	9. *Fusobacterium hwasookii* ChDC F206, complete genome CP013336.1	96%	83%
	10. *Propionigenium maris* 23S rRNA gene, strain DSM 9537 T AJ307979.1	93%	83%
2 (1376 bp)	1. *Sneathia amnii* strain SN35, complete genome CP011280.1	99%	93%
	2. Uncultured bacterium clone T21VE9_4 16S ribosomal RNA gene, partial sequence KR514404.1	67%	100%
	3. Uncultured bacterium clone T21UE13_2 16S ribosomal RNA gene, partial sequence KR514401.1	67%	100%
	4. Uncultured bacterium clone T21VE9_24 16S ribosomal RNA gene, partial sequence KR514494.1	67%	99%
	5. Uncultured bacterium clone T21VE9_36 16S ribosomal RNA gene, partial sequence KR514405.1	67%	99%
	6. Uncultured bacterium clone T21UE13_23 16S ribosomal RNA gene, partial sequence KR514403.1	67%	99%
	7. Uncultured bacterium clone T21UE13_12 16S ribosomal RNA gene, partial sequence KR514402.1	67%	99%
	8. Uncultured bacterium clone H94 16S ribosomal RNA gene, partial sequence KC894542.1	69%	99%
	9. Uncultured bacterium clone H35 16S ribosomal RNA gene, partial sequence KC894531.1	69%	99%
	10. Uncultured bacterium clone T21UE060212_22 16S ribosomal RNA gene, partial sequence KR514400.1	67%	99%
3 (744 bp)	1. *Histophilus somni* strain 2336 23S ribosomal RNA gene, complete sequence NR_103965.1	100%	99%
	2. *Histophilus somni* strain 129P 23S ribosomal RNA gene, complete sequence NR_076444.1	100%	99%
	3. *Haemophilus somnus* 2336, complete genome CP000947.1	100%	99%
	4. *Haemophilus somnus* 129PT, complete genome CP000436.1	100%	99%
	5. *Pasteurella multocida* subsp. *multocida* OH4807, complete genome CP004391.1	100%	94%
	6. *Pasteurella multocida* subsp. *multocida* str. HN06, complete genome CP003313.1	100%	94%
	7. *Pasteurella multocida* subsp. *multocida* PMTB2.1, complete genome CP007205.1	100%	94%
	8. *Pasteurella multocida subsp. multocida* strain Pm70 23S ribosomal RNA gene, complete sequence NR_103956.1	100%	94%
	9. *Pasteurella multocida subsp. multocida* str. Pm70, complete genome AE004439.1	100%	94%
	10. *Pasteurella multocida strain* Pm-3, complete genome CP014618.1	100%	94%

Database = Non redundant (nr) Nucleotide collection (nt).

Program = BLASTN 2.3.0+ [45, 46]

## Discussion

At the time of writing there was a paucity of reports on high throughput 16S amplicon sequencing and metagenomic shotgun sequencing in lung tissue in cattle and sheep. A detailed recent study was conducted on the lung microbiome in sheep [42] but, to our knowledge, the present report is the first high throughput 16S amplicon sequencing study in lung and corresponding mediastinal lymph nodes in multiple cattle with BRD. The cranial lung lobe tissue was chosen for investigation as it is the most common site of BRD lesions [43, 44]. Mediastinal lymph node tissue was also examined as respiratory disease causing viruses and bacteria often disseminate to these lymph nodes from the lungs [47–50]. To gain an understanding of the bacteria associated with BRD cases in Ireland, we analysed lung tissue from ‘real’ BRD fatalities on a number of farms in three different regions of the country. Obtaining lung and lymph node tissue from healthy ‘matched’ controls (i.e. same breed, sex, sire, age, farm) was not feasible in this study as it would have required slaughtering a healthy animal on the same farm as every BRD case. Instead, we had access to healthy calves which were being slaughtered as part of another study from which we took lung and lymph node tissue. In the present study, the composition of bacteria in the lungs and lymph nodes were very different between animals. In several cases, bacterial genera were different in lung and mediastinal lymph node tissues from the same calf.

In humans, the lungs of clinically healthy individuals were originally thought to be sterile [51]. However, recent studies have demonstrated that small numbers of bacteria inhabit the lungs of clinically healthy humans and sheep [44, 51]. The current study also indicated that there are low numbers of bacteria in the lungs and additionally in the lymph nodes of clinically healthy calves. *Prevotella* was the most prevalent genus in terms of relative abundance in the lesion-free lungs but in terms of raw read counts, similar numbers of reads were detected in fatal BRD cases. This is a highly abundant anaerobic genus in the rumen so unlikely to actively grow in aerobic lung tissue and probably passes into the upper regions of the lung by inhalation when rumen regurgitation occurs. A possible explanation for the greater number of bacterial reads found in fatal cases of BRD compared with the clinically healthy calves (with and without lung lesions) in both tissues, is that the disease may cause a proliferation of bacteria that can overcome the natural “equilibrium” in healthy individuals.

Although the 20 H-F calves in the “clinically healthy” group displayed no clinical signs of disease at slaughter and only 5 of these calves had been previously treated for BRD, lung lesions were observed in 12 of these 20 animals (60%). This is similar to observations by Schneider et al. [52] and Wittum et al. [53], who reported that 61% and 68%, of feedlot steers with no recorded history of BRD presented with lung lesions at slaughter, respectively [53]. These authors suggested that many cases of BRD can be missed or may be sub-clinical [52].

*Leptotrichiaceae, Fusobacterium*, *Mycoplasma, Trueperella* and *Bacteroides* were more abundant among post-mortem lung tissue samples from dairy calves which died from BRD compared to the lesion-free lung tissue samples from clinically healthy H-F calves. Additionally, *Leptotrichiaceae*, *Mycoplasma* and *Pasteurellaceae* had significantly greater relative abundances among post-mortem lymph node tissue samples from dairy calves which died from BRD relative to the clinically healthy, lung lesion-free H-F calves. It is also possible that other bacterial OTUs were more abundant and present more often within tissue samples from BRD-affected relative to clinically healthy dairy calves, however, as this study lacked power due to restricted sample sizes of BRD-affected dairy calves and lung lesion-free, clinically healthy dairy calves, these differences were not statistically different.

It is possible that the bacterial OTUs present among the samples obtained from the RVLs were the result of contaminating bacteria which propagated post-mortem. However, this is unlikely as most bacterial OTUs found among these samples were also found in the samples from the clinically healthy calves with lung lesions, in which the samples were obtained immediately following slaughter.

### Leptotrichiaceae

The *Leptotrichiaceae* OTU was more abundant within post-mortem lung and lymph node tissue samples from dairy calves which died from BRD relative to post-mortem lung and lymph node tissue samples from clinically healthy, lung lesion-free, H-F calves. Additionally, it was one of the most abundant OTUs identified within tissue samples from beef and dairy calves which died from BRD and was absent in samples from clinically healthy H-F calves which had no lung lesions.

The prevalence of OTUs that were classified as the bacterial family *Leptotrichiaceae* in lung and lymph node tissue from fatal BRD cases and lung lesions from clinically healthy cattle was of particular interest as members of this family were recently proposed to be associated with lung disease in humans. *Leptotrichiaceae* species are not currently associated with BRD and have not been previously described in lungs or mediastinal lymph nodes of cattle. All members of this bacterial family are facultative to obligate anaerobic Gram-negative rods [54]. They can occur in anoxic environments as well as oral and intestinal environments [54]. Although little is known about their role in disease, mainly due to the difficulties associated with their isolation and identification, they have been suggested as emerging pathogens [55]. A member of this family within the genus *Leptotrichia* was identified in bronchoalveolar lavage fluid from an elderly man with pneumonia and was hypothesized to be responsible for the disease [56]. Additionally, a member of the genus *Sneathia* was hypothesised to be responsible for late-onset bronchiolitis obliterans syndrome in a lung transplant recipient [57]. Furthermore, species within the *Leptotrichia* genus have been isolated from blood cultures of patients with lesions in the oral mucosa [55] and from blood cultures from patients with anaerobic bloodstream infection receiving high-dose chemotherapy [58].

As the OTU sequence for *Leptotrichiaceae* found in the present study was not identical to any known genus we wanted to achieve better classification with longer DNA sequences than was possible with the 472 bp V3-V4 16S sequence. To do this we partially sequenced the genome of this unknown species of bacteria by metagenomic shotgun sequencing and de novo assembly of a bacterial DNA enriched post-mortem lung tissue sample. From this we obtained three bacterial sequence contigs which were 2647 bp, 1376 bp and 744 bp long. The 744 bp contig corresponded to a known BRD-associated bacterial species, *Histophilus somni*. A BLAST search against the NCBI cultured bacteria database showed the 2647 bp and 1376 bp contigs were most similar (92 and 93% identity respectively) to *Sneathia amnii.* A BLAST search against the nr/nt collection showed that part of one of the *Leptotrichiaceae* contigs (67%) was 100% identical to an uncultured clone. As this uncultured clone (GenBank: KR514404.1, submitted by Lu, J) was obtained from the bovine reproductive tract of farm animals at University College Dublin, it appears to be a novel species that is present in at least two microbiomes in cattle. From the present study it is not possible to infer whether this novel *Leptotrichiaceae* species is pathogenic and causing lung lesions in cattle or wheteher it is merely able to grow opportunistically in the relatively anaerobic lung lesions (some *Leptotrichiaceae* are facultative anaerobes) which have been caused by known pathogens. In lung tissue samples from the calves in this study, the *Leptotrichiaceae* OTU always co-occurred with either *Pasteurellaceae, Mycoplasma* or *Fusobacterium* OTUs.

### Pasteurellaceae

*Pasteurellaceae* was one of the most abundant OTUs identified overall and was consistently more abundant in post-mortem lymph node samples from dairy calves which died from BRD compared with those from clinically healthy, lung lesion-free, H-F calves. Many bacterial species implicated in BRD, including *Mannheimia haemolytica, Histophilus somni, Bibersteinia trehalosi* and *Pasteurella multocida*, belong to the *Pasteurellaceae* family. Indeed, these bacterial species possess many virulence factors which enable them to become pathogenic [17–19, 38, 59, 60]. Furthermore they have been commonly isolated from the lungs and respiratory tracts of cattle with BRD and healthy cattle [11, 17, 19, 22, 61–64].

### Fusobacterium

*Fusobacterium* was one of the most abundant OTUs identified. Furthermore, within the post mortem lung tissue samples, it was found to be more abundant among samples from dairy calves which died from BRD relative to the samples from the clinically healthy, lung lesion-free, H-F calves. Additionally, it was not present within any of the tissue samples from the calves that had no lung lesions. This was not surprising as *Fusobacterium* species are commonly isolated from chronic, abscessing lung lesions in cattle with BRD [11]. Furthermore, this genus contains the anaerobic species *Fusobacterium necrophorum*, which is a significant opportunistic animal pathogen with several virulence factors [65] and it has been previously isolated from ruminant respiratory tracts [63, 66] and pneumonic bovine lungs [67].

### Mycoplasma

*Mycoplasma* was one of the most abundant OTUs present in the post-mortem lung and lymph node samples. It was found to be more abundant among the tissue samples from dairy calves which died from BRD relative to the tissue samples from the clinically healthy, lung lesion-free, H-F calves. This is consistent with previous studies which reported *Mycoplasma* to be one of the dominant genera in nasopharyngeal swab samples from cattle at feedlot [26, 33], despite being infrequent in cattle at feedlot entry [26]. Additionally, this result is concordant with previous observations which report that *Mycoplasma* species *bovis, dispar* and *bovirhinis,* were identified more often in pneumonic lungs and respiratory tracts compared with clinically healthy lungs and respiratory tracts [25, 62, 68, 69].

Although *Mycoplasma bovis* is a recognised BRD pathogen [70] and is commonly screened for in veterinary diagnostic laboratories [71, 72], the other major *Mycoplasma* species are not generally screened. However, they may be responsible for BRD as *Mycoplasma dispar* is a recognised pathogenic *Mycoplasma* species [10], capable of colonising the lower respiratory tract and caused pneumonia when inoculated into gnotobiotic calves [73]. Moreover, it has been cultured from the respiratory tracts of calves presenting with BRD [63, 74] and has been isolated from lavage fluids of calves with recurrent respiratory disease [68]. Furthermore, although *Mycoplasma arginine* and *Mycoplasma bovirhinis* did not cause pneumonia following inoculation into gnotobiotic calves, these species have also been isolated from lavage fluids of calves with recurrent respiratory disease [68] and pneumonic lungs [68, 69] and have been suggested to act as co-pathogens which may intensify respiratory disease symptoms [69, 74].

### Ureaplasma

*Ureaplasmas*, also belong to the same family as the *Mycoplasmas,* and are pathogenic bacteria which were initially associated with urogenital tract infections but have also been isolated from pneumonic bovine lungs [10, 68, 75]. The species *Ureaplasma diversum* has been associated with clinical respiratory disease [75, 76]. As *Ureaplasma* was found to be present in pneumonic lung tissue samples from calves which died from BRD, this genus may be an important contributor to BRD which is often overlooked.

### Bacterioides

*Bacterioides* species are associated with bacterial pneumonia [1, 18]. They had high relative abundances within the post-mortem lung and lymph node tissue samples taken from beef and dairy calves which died from BRD. Furthermore, they were more abundant among samples from dairy calves which died from BRD compared with clinically healthy lesion-free dairy calves. This result was expected as *Bacterioides* species have been commonly isolated from chronic, abscessing lung lesions in cattle with BRD [11] and have previously been cultured from pneumonic bovine lungs [67].

### NGS 16S compared to RVL results

The bacterial 16S rRNA gene amplicon sequencing assay identified many more bacteria from calves which died from BRD than the culture and PCR tests carried out at the RVLs. However, at the RVLs, there were several cases where bacterial species were identified by culture, and one case where a bacterial species was identified by PCR, which were not identified by the bacterial 16S rRNA gene amplicon sequencing assay. This may be because only a small part of the cranial lobe region of the lung and the mediastinal lymph node were examined with the 16S sequencing assay, whereas culture was performed on swab samples covering the whole lung area at RVLs. In the present study, the 16S rRNA gene amplicon assay showed that many bacteria which are not currently screened for by PCR or cultured at RVLs were found to be present within the cranial lung lobes and mediastinal lymph nodes from calves which died from BRD, including *Leptotrichiaceae, Fusobacterium, Helcococcus, Mycoplasma, Ureaplasma,* and *Bacteroides*.

Although *Mycoplasma bovis* is currently screened for by PCR in RVLs, the bacterial 16S rRNA gene amplicon sequencing assay found the *Mycoplasma* OTU to be present in seven calves which tested negative for *Mycoplasma bovis* by PCR at RVLs. This is most likely because other species belonging to the *Mycoplasma* genus, other than *Mycoplasma bovis*, were present within these samples. Despite *Helcococcus* species not being currently associated with BRD, *Helcococcus ovis* has been isolated from sheep with respiratory disease [77], a calf with valvular endocarditis [78] and from a horse with a pulmonary abscess [79]. Therefore, implementation of this 16S rRNA amplicon sequencing assay at veterinary diagnostic laboratories would enable discovery of more of the bacteria responsible for each case of BRD as the present study has highlighted that this assay can identify more known bacteria (both culturable and non-culturable) than culture, and can also identify yet unknown and unculturable bacteria in tissue samples, which cannot be identified by culture and PCR tests currently carried out at veterinary diagnostic laboratories.

Next generation sequencing can revolutionise BRD bacterial diagnostics. By using a single 16S rRNA amplicon sequencing assay, the bacteria present in a tissue sample can be accurately identified. A limitation of the Illumina MiSeq based bacterial 16S rRNA gene amplicon sequencing assay is that currently, it is unable to identify bacterial OTUs to species level due to the fact that the maximum read length is <550 base pairs. However, the entire 16S rRNA gene can be sequenced with Pacific Biosciences’ single molecule, real-time sequencing technology but this is expensive and the error rate for individual reads are high [80].

## Conclusions

In conclusion, we have shown that a convenient, single universal bacterial 16S rRNA gene amplicon sequencing assay can detect the bacterial OTUs present in a BRD-affected lung or lymph node sample. Using this assay, we have characterised the microbiomes of the cranial lung lobe and mediastinal lymph node from calves which died from BRD and from clinically healthy H-F calves. We have confirmed that the cranial region of bovine lungs are not sterile environments and that some bacteria associated with BRD can be present within the lung and lymph node tissues of clinically healthy calves as well as those calves which were suffering from BRD. However, the frequency of the detection of bacteria associated with BRD is lower in the lungs and lymph nodes of clinically healthy calves compared with calves which died from BRD.

Furthermore, we have identified bacterial sequences corresponding to an unidentified species, which is classified as a member of the bacterial family *Leptotrichiaceae,* that was a dominant species in bovine lung lesions in BRD cases and absent in healthy lesion-free lung tissue. Therefore, this 16S rRNA gene amplicon sequencing assay has potential to expedite BRD diagnosis and identify as of yet unknown bacteria which may be key players in BRD development and progression. Furthermore, results from the 16S rRNA gene amplicon sequencing assay, presented in this paper, highlight that continually focusing on bacterial agents that have been commonly associated with BRD could lead to common commensals that may offer a pathogenic threat being neglected. This could result in increased morbidity, increased mortality, and decreased performance in beef and dairy calves.

The present study shows that further NGS study of large numbers of healthy and BRD lung and lymph node tissue samples to generate a comprehensive view of the microbiome of healthy and diseased bovine lungs is warranted.

## Additional files

Additional file 1: Description of post-mortem tissue samples collected, sample preservation methods and the diagnostics tests performed on these samples at regional veterinary laboratories (RVL)s. (XLSX 18 kb)

Additional file 2: Clinical assessment report from the clinically healthy calves on the morning before slaughter. (DOCX 31 kb)

Additional file 3: Description of the lungs from the clinically healthy calves observed following slaughter. (DOCX 14 kb)

Additional file 4: Agilent 2100 bioanalyzer DNA Electrophoresis assay results (DOCX 222 kb)

Additional file 5: Operational taxonomic unit absolute read counts table. (XLSX 5305 kb)

Additional file 6: Operational taxonomic unit relative abundance table. (XLSX 78 kb)

Additional file 7: Comparison of OTU abundance between post-mortem lung tissue samples from dairy calves which died from BRD (*n* = 6) and clinically healthy calves without lung lesions (*n* = 8). (DOCX 35 kb)

Additional file 8: Comparison of OTU abundance between post-mortem lymph node tissue samples from dairy calves which died from BRD (*n* = 5) and clinically healthy calves without lung lesions (*n* = 8). (DOCX 33 kb)

Additional file 9: Description of the results of the PCR and culture tests carried out at the regional veterinary laboratories and the corresponding results of the OTUs identified by 16S rRNA amplicon sequencing. (DOCX 24 kb)

Additional file 10: Assembled bacterial contig sequences. (DOCX 30 kb)

## Abbreviations

BCoV: Bovine coronavirus
Bo-HV1: Bovine herpesvirus 1
BPIV-3: Bovine parainfluenza 3 virus
BRD: Bovine respiratory disease
BRSV: Bovine respiratory syncytial virus
BVDV: Bovine viral diarrhoea virus
H-F: Holstein-Friesian
NGS 16S amplicon analysis: Next generation sequencing of 16S ribosomal RNA gene PCR amplicons
OTU: Operational taxonomic unit
QIIME: Quantitative insights into microbial ecology
rRNA: Ribosomal RNA
RVL: Regional veterinary laboratory
