# Supplementary material for: Illumina MiSeq 16S amplicon sequence analysis of bovine respiratory disease associated bacteria in lung and mediastinal lymph node tissue
Source: BMC Vet Res. 2017 May 2;13:118. doi: 10.1186/s12917-017-1035-2 (PMC5414144; doi:10.1186/s12917-017-1035-2)
Supplement: Supplementary file 2 — Clinical assessment report from the clinically healthy calves on the morning before slaughter. (DOCX 31 kb) [file 12917_2017_1035_MOESM2_ESM.docx]

**Additional file 2.** **Clinical assessment report from the healthy Holstein-Friesian calves on the morning before slaughter.**

| **Calf I.D.** | **Eye score** | **Ear score** | **Nasal score** | **Rectal temp** | **Cough** | **Weight (kg)** | **Age (days)** | **Respiratory score** |
| --- | --- | --- | --- | --- | --- | --- | --- | --- |
| **1** | 0 | 0 | 0 | 38.2 | 0 | 92.0 | 92 | 0 |
| **2** | 0 | 0 | 1 | 38.3 | 0 | 91.0 | 93 | 2 |
| **3** | 0 | 0 | 0 | 38.4 | 0 | 104.5 | 87 | 1 |
| **4** | 0 | 0 | 0 | 39.0 | 0 | 84.5 | 87 | 2 |
| **5** | 0 | 0 | 0 | 38.5 | 0 | 95.0 | 90 | 1 |
| **6** | 0 | 0 | 0 | 39.0 | 0 | 99.0 | 86 | 2 |
| **7** | 1 | 0 | 0 | 38.9 | 0 | 93.0 | 91 | 3 |
| **8** | 1 | 0 | 0 | 38.4 | 0 | 92.0 | 88 | 2 |
| **9** | 0 | 0 | 2 | 38.5 | 0 | 86.0 | 90 | 4 |
| **10** | 0 | 0 | 1 | 38.4 | 1 | 86.5 | 91 | 3 |
| **11** | 0 | 0 | 0 | 38.6 | 0 | 98.5 | 88 | 1 |
| **12** | 0 | 0 | 0 | 38.4 | 0 | 93.0 | 91 | 1 |
| **13** | 1 | 0 | 2 | 38.3 | 0 | 90.0 | 95 | 4 |
| **14** | 0 | 0 | 0 | 38.5 | 0 | 100.5 | 92 | 1 |
| **15** | 1 | 0 | 0 | 38.7 | 0 | 82.5 | 82 | 2 |
| **16** | 0 | 0 | 0 | 38.1 | 0 | 82.0 | 72 | 0 |
| **17** | 2 | 0 | 0 | 38.2 | 0 | 82.5 | 75 | 2 |
| **18** | 0 | 0 | 0 | 38.0 | 0 | 83.5 | 74 | 0 |
| **19** | 0 | 0 | 1 | 39.1 | 0 | 77.5 | 77 | 3 |
| **20** | 0 | 0 | 0 | 38.5 | 0 | 82.5 | 89 | 1 |

Eye, ear, nasal, cough and respiratory score were classified using the Wisconsin calf health scoring criteria (<https://www.vetmed.wisc.edu/dms/fapm/fapmtools/8calf/calf_respiratory_scoring_chart.pdf>).
