# Supplementary material for: Illumina MiSeq 16S amplicon sequence analysis of bovine respiratory disease associated bacteria in lung and mediastinal lymph node tissue
Source: BMC Vet Res. 2017 May 2;13:118. doi: 10.1186/s12917-017-1035-2 (PMC5414144; doi:10.1186/s12917-017-1035-2)
Supplement: Supplementary file 3 — Description of the lungs from the clinically healthy calves observed following slaughter. (DOCX 14 kb) [file 12917_2017_1035_MOESM3_ESM.docx]

**Additional file 3.** **Description of the lungs from the clinically healthy calves observed following slaughter.**

| **Calf I.D.** | **No. incidents of BRD** | **Description of lungs following slaughter** |
| --- | --- | --- |
| 1 | 0 | Lesions present on a small piece of the middle lobe |
| 2 | 0 | Lesions present on one cranial lobe |
| 3 | 0 | Clinically healthy lungs |
| 4 | 0 | Lesions present on part of the left caudal lobe and a small part of the left middle lobe |
| 5 | 0 | Clinically healthy lungs |
| 6 | 0 | Clinically healthy lungs |
| 7 | 0 | Clinically healthy lungs |
| 8 | 0 | Clinically healthy lungs |
| 9 | 0 | Lesions present on one cranial lobe |
| 10 | 1 | Lesions present on one cranial lobe |
| 11 | 0 | Small lesions present on one cranial lobe |
| 12 | 0 | Small lesions present on cranial and middle lobes |
| 13 | 0 | Clinically healthy lungs |
| 14 | 0 | Clinically healthy lungs |
| 15 | 2 | Lesions present on cranial lobe |
| 16 | 1 | Lesions present on both cranial lobes and lesions present on middle lobe |
| 17 | 1 | Lesions present on cranial lobe |
| 18 | 0 | Lesion present on middle lobe |
| 19 | 0 | Clinically healthy lungs |
| 20 | 2 | Lesions present on cranial lobe |

Incidents of BRD refers to incidents when a calf was diagnosed with BRD and treated with antibiotics.

Lesions were defined as macroscopic consolidation or abscessation of lung tissue.
