# Supplementary material for: Illumina MiSeq 16S amplicon sequence analysis of bovine respiratory disease associated bacteria in lung and mediastinal lymph node tissue
Source: BMC Vet Res. 2017 May 2;13:118. doi: 10.1186/s12917-017-1035-2 (PMC5414144; doi:10.1186/s12917-017-1035-2)
Supplement: Supplementary file 4 — Agilent 2100 bioanalyzer DNA Electrophoresis assay results (DOCX 222 kb) [file 12917_2017_1035_MOESM4_ESM.docx]

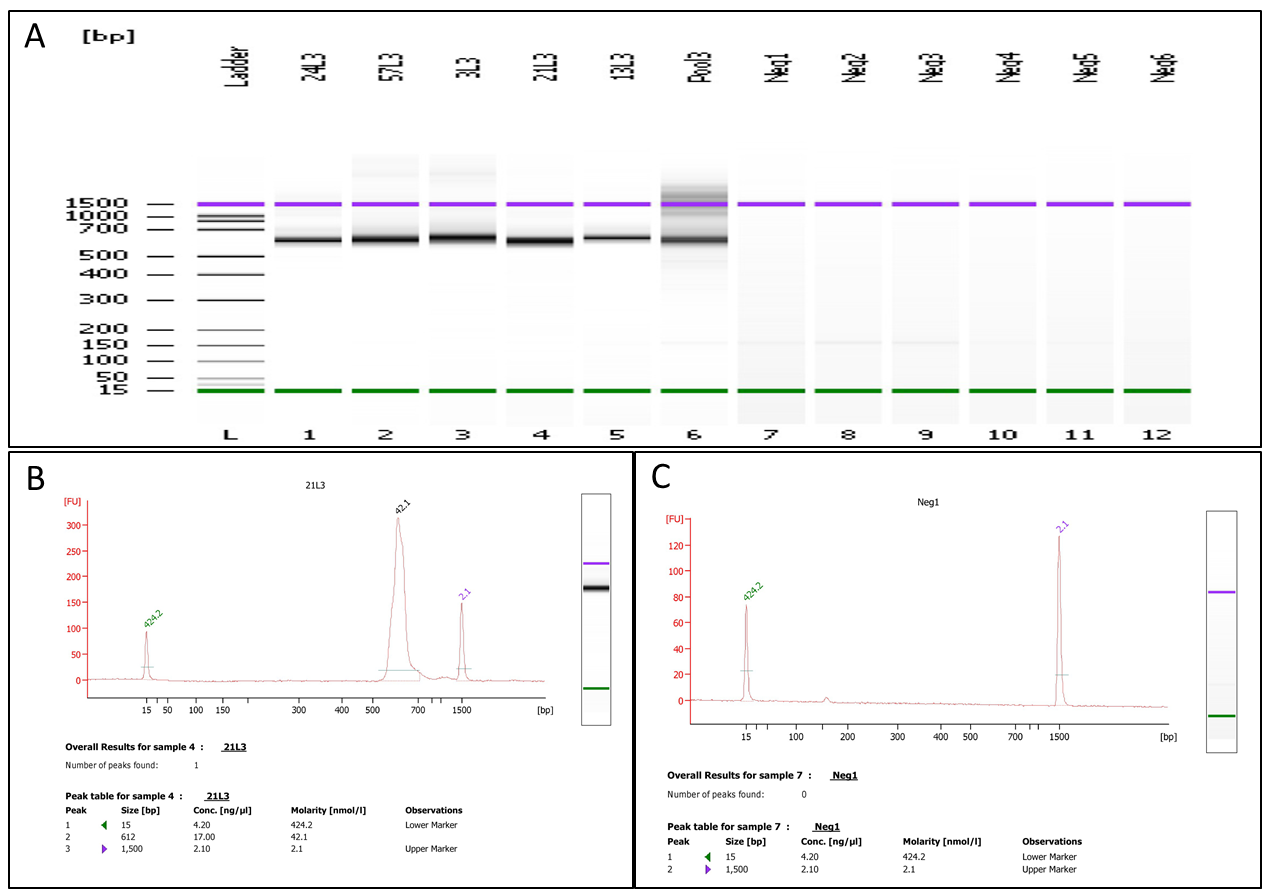


**Additional file 4.** **Agilent 2100 bioanalyzer DNA Electrophoresis assay results.** A = Gel image of the DNA ladder and 12 samples (samples 1-5 are lung tissue DNA libraries, sample 6 is a pool of all libraries, samples 7-12 are the negative controls). B = Electropherogram summary for an individual lung tissue library. C = Electropherogram summary for an individual negative control (H_2_O) library.
