# Supplementary material for: Illumina MiSeq 16S amplicon sequence analysis of bovine respiratory disease associated bacteria in lung and mediastinal lymph node tissue
Source: BMC Vet Res. 2017 May 2;13:118. doi: 10.1186/s12917-017-1035-2 (PMC5414144; doi:10.1186/s12917-017-1035-2)
Supplement: Supplementary file 7 — Comparison of OTU abundance between post-mortem lung tissue samples from dairy calves which died from BRD (n = 6) and clinically healthy calves without lung lesions (n = 8). (DOCX 35 kb) [file 12917_2017_1035_MOESM7_ESM.docx]

**Additional file 7. Comparison of OTU abundance between post-mortem lung tissue samples from dairy calves which died from BRD (n = 6) and clinically healthy calves without lung lesions (n = 8).**

| **OTU** | **Mean relative abundance**  **(dairy calves which died from BRD)** | **S.E.M** | **Mean relative abundance**  **(clinically healthy)** | **S.E.M** | **Mann-Whitney U** | **P value** |
| --- | --- | --- | --- | --- | --- | --- |
| ***Trueperella*** | 0.55 | 0.45 | 0.00 | 0.00 | 8.00 | **0.02** |
| ***Microbacteriaceae*** | 0.02 | 0.01 | 0.00 | 0.00 | 16.00 | 0.16 |
| ***Rothia*** | 0.00 | 0.00 | 0.15 | 0.15 | 21.00 | > 0.99 |
| ***Bifidobacterium*** | 0.01 | 0.01 | 0.00 | 0.00 | 20.00 | 0.43 |
| ***Coriobacteriaceae*** | 0.02 | 0.02 | 0.25 | 0.24 | 22.00 | 0.85 |
| ***Bacteroidales*** | 0.03 | 0.03 | 1.17 | 0.67 | 14.00 | 0.13 |
| ***BS11*** | 0.00 | 0.00 | 0.23 | 0.15 | 18.00 | 0.47 |
| ***5-7N15*** | 0.00 | 0.00 | 0.55 | 0.37 | 18.00 | 0.47 |
| ***Bacteroides*** | 8.10 | 6.00 | 7.38 | 7.38 | 9.50 | **0.03** |
| ***Porphyromonas*** | 0.37 | 0.32 | 0.00 | 0.00 | 12.00 | 0.05 |
| ***RF16*** | 0.24 | 0.24 | 0.04 | 0.03 | 23.00 | > 0.99 |
| ***S24-7*** | 0.06 | 0.06 | 5.97 | 5.24 | 21.00 | 0.69 |
| ***Paraprevotellaceae*** | 0.00 | 0.00 | 0.04 | 0.03 | 18.00 | 0.47 |
| ***CF231*** | 0.03 | 0.03 | 0.02 | 0.02 | 22.50 | 0.74 |
| ***Prevotella (total)*** | 0.79 | 0.79 | 30.67 | 7.56 | 1.00 | **<0.01** |
| ***p-2534-18B5*** | 0.00 | 0.00 | 0.12 | 0.12 | 21.00 | > 0.99 |
| ***Chryseobacterium*** | 0.00 | 0.00 | 0.25 | 0.25 | 21.00 | > 0.99 |
| ***Cloacibacterium*** | 0.01 | 0.01 | 0.95 | 0.95 | 23.50 | > 0.99 |
| ***Chitinophagaceae*** | 0.17 | 0.17 | 0.00 | 0.00 | 20.00 | 0.43 |
| ***Chlamydia*** | 0.00 | 0.00 | 0.00 | 0.00 | 20.00 | 0.43 |
| ***YS2*** | 0.00 | 0.00 | 0.44 | 0.44 | 21.00 | > 0.99 |
| ***Fibrobacter*** | 0.21 | 0.21 | 0.47 | 0.47 | 23.50 | > 0.99 |
| ***Bacillus*** | 0.00 | 0.00 | 0.25 | 0.25 | 21.00 | > 0.99 |
| ***Lysinibacillus*** | 0.06 | 0.06 | 0.85 | 0.85 | 23.50 | > 0.99 |
| ***Granulicatella*** | 0.00 | 0.00 | 1.56 | 1.56 | 21.00 | > 0.99 |
| ***Lactobacillus*** | 0.01 | 0.01 | 0.00 | 0.00 | 20.00 | 0.43 |
| ***Streptococcus*** | 0.06 | 0.06 | 0.66 | 0.45 | 17.50 | 0.33 |
| ***Clostridiales*** | 0.00 | 0.00 | 4.21 | 2.09 | 17.50 | 0.33 |
| ***Christensenellaceae*** | 0.00 | 0.00 | 0.07 | 0.07 | 23.50 | > 0.99 |
| ***Clostridiaceae*** | 0.00 | 0.00 | 0.00 | 0.00 | 20.00 | 0.43 |
| ***Clostridium*** | 2.30 | 1.70 | 5.50 | 3.78 | 19.00 | 0.54 |
| ***Lachnospiraceae*** | 0.06 | 0.05 | 0.95 | 0.85 | 22.00 | 0.79 |
| ***Butyrivibrio*** | 0.05 | 0.05 | 1.43 | 0.74 | 17.50 | 0.33 |
| ***Coprococcus*** | 0.02 | 0.02 | 0.00 | 0.00 | 20.00 | 0.43 |
| ***Pseudobutyrivibrio*** | 0.00 | 0.00 | 0.23 | 0.23 | 21.00 | > 0.99 |
| ***Shuttleworthia*** | 0.00 | 0.00 | 0.11 | 0.11 | 21.00 | > 0.99 |
| ***Ruminococcaceae*** | 0.10 | 0.10 | 3.59 | 2.56 | 18.50 | 0.41 |
| ***Ruminococcus*** | 0.01 | 0.01 | 0.22 | 0.18 | 17.50 | 0.33 |
| ***Veillonellaceae*** | 0.00 | 0.00 | 4.40 | 4.17 | 18.00 | 0.47 |
| ***Acidaminococcus*** | 0.05 | 0.05 | 0.00 | 0.00 | 20.00 | 0.43 |
| ***Dialister*** | 0.00 | 0.00 | 0.19 | 0.19 | 21.00 | > 0.99 |
| ***Megasphaera*** | 0.07 | 0.07 | 0.00 | 0.00 | 20.00 | 0.43 |
| ***Phascolarctobacterium*** | 0.00 | 0.00 | 3.99 | 3.93 | 15.00 | 0.21 |
| ***Selenomonas*** | 0.01 | 0.01 | 0.00 | 0.00 | 20.00 | 0.43 |
| ***Succiniclasticum*** | 0.19 | 0.19 | 2.43 | 1.19 | 14.00 | 0.13 |
| ***Veillonella*** | 0.01 | 0.01 | 0.46 | 0.46 | 23.50 | > 0.99 |
| ***Mogibacteriaceae*** | 0.00 | 0.00 | 0.00 | 0.00 | 20.00 | 0.43 |
| ***Helcococcus*** | 0.01 | 0.01 | 0.00 | 0.00 | 16.00 | 0.16 |
| ***Eubacterium*** | 0.00 | 0.00 | 0.06 | 0.06 | 21.00 | > 0.99 |
| ***Fusobacterium*** | 15.00 | 8.70 | 0.00 | 0.00 | 8.00 | **0.02** |
| ***Leptotrichiacea*** | 26.00 | 14.00 | 0.00 | 0.00 | 0.00 | **<0.01** |
| ***Pirellulaceae*** | 0.01 | 0.01 | 0.34 | 0.32 | 21.00 | 0.69 |
| ***Agrobacterium*** | 0.02 | 0.02 | 0.01 | 0.01 | 22.50 | 0.74 |
| ***Anaplasma*** | 0.03 | 0.03 | 0.00 | 0.00 | 20.00 | 0.43 |
| ***Sphingomonas*** | 0.11 | 0.11 | 0.00 | 0.00 | 20.00 | 0.43 |
| ***Tepidimonas*** | 0.00 | 0.00 | 0.74 | 0.74 | 21.00 | > 0.99 |
| ***Campylobacter*** | 0.11 | 0.10 | 0.00 | 0.00 | 16.00 | 0.16 |
| ***Succinivibrionaceae*** | 0.13 | 0.13 | 5.13 | 3.14 | 14.00 | 0.13 |
| ***Ruminobacter*** | 0.00 | 0.00 | 0.43 | 0.43 | 21.00 | > 0.99 |
| ***Enterobacteriaceae*** | 0.65 | 0.51 | 0.00 | 0.00 | 12.00 | 0.05 |
| ***Proteus*** | 0.11 | 0.11 | 0.00 | 0.00 | 20.00 | 0.43 |
| ***Pasteurellaceae (total)*** | 16.00 | 7.40 | 6.58 | 5.03 | 15.00 | 0.25 |
| ***Actinobacillus*** | 0.43 | 0.33 | 1.26 | 1.26 | 20.00 | 0.54 |
| ***Pasteurella*** | 1.40 | 1.40 | 0.00 | 0.00 | 12.00 | 0.05 |
| ***Acinetobacter*** | 0.00 | 0.00 | 0.00 | 0.00 | 20.00 | 0.43 |
| ***Psychrobacter*** | 0.04 | 0.04 | 0.00 | 0.00 | 20.00 | 0.43 |
| ***Xanthomonadaceae*** | 0.00 | 0.00 | 0.06 | 0.06 | 21.00 | > 0.99 |
| ***Treponema*** | 0.03 | 0.03 | 0.23 | 0.23 | 23.50 | > 0.99 |
| ***F16*** | 0.05 | 0.05 | 0.17 | 0.11 | 21.00 | 0.69 |
| ***Rs-045*** | 0.00 | 0.00 | 1.63 | 1.63 | 21.00 | > 0.99 |
| ***Mycoplasma*** | 22.00 | 11.00 | 3.58 | 3.52 | 4.00 | **0.01** |
| ***Ureaplasma*** | 4.50 | 4.40 | 0.00 | 0.00 | 12.00 | 0.05 |
| ***RFP12*** | 0.00 | 0.00 | 0.00 | 0.00 | 20.00 | 0.43 |
| ***WPS-2*** | 0.01 | 0.01 | 0.00 | 0.00 | 20.00 | 0.43 |
