# Supplementary material for: Illumina MiSeq 16S amplicon sequence analysis of bovine respiratory disease associated bacteria in lung and mediastinal lymph node tissue
Source: BMC Vet Res. 2017 May 2;13:118. doi: 10.1186/s12917-017-1035-2 (PMC5414144; doi:10.1186/s12917-017-1035-2)
Supplement: Supplementary file 8 — Comparison of OTU abundance between post-mortem lymph node tissue samples from dairy calves which died from BRD (n = 5) and clinically healthy calves without lung lesions (n = 8). (DOCX 33 kb) [file 12917_2017_1035_MOESM8_ESM.docx]

**Additional file 8. Comparison of OTU abundance between post-mortem lymph node tissue samples from dairy calves which died from BRD (n = 5) and clinically healthy calves without lung lesions (n = 8).**

| **OTU** | **Mean relative abundance**  **(dairy calves which died from BRD)** | **S.E.M** | **Mean relative abundance**  **(clinically healthy)** | **S.E.M** | **Mann-Whitney U** | **P value** |
| --- | --- | --- | --- | --- | --- | --- |
| ***Trueperella*** | 0.65 | 0.58 | 0.00 | 0.00 | 12.00 | 0.13 |
| ***Microbacteriaceae*** | 0.01 | 0.01 | 0.00 | 0.00 | 16.00 | 0.38 |
| ***Arthrobacter*** | 0.00 | 0.00 | 0.61 | 0.61 | 17.50 | > 0.99 |
| ***Bifidobacterium*** | 0.06 | 0.06 | 0.00 | 0.00 | 16.00 | 0.38 |
| ***Coriobacteriaceae*** | 0.01 | 0.01 | 0.08 | 0.08 | 19.00 | > 0.99 |
| ***Bacteroidales*** | 0.00 | 0.00 | 2.78 | 1.85 | 12.50 | 0.23 |
| ***Bacteroides*** | 7.42 | 4.54 | 9.96 | 9.96 | 15.50 | 0.51 |
| ***Porphyromonas*** | 1.84 | 1.84 | 0.00 | 0.00 | 16.00 | 0.38 |
| ***RF16*** | 0.00 | 0.00 | 0.48 | 0.42 | 15.00 | 0.49 |
| ***S24-7*** | 0.00 | 0.00 | 0.01 | 0.01 | 17.50 | > 0.99 |
| ***Paraprevotellaceae*** | 0.00 | 0.00 | 0.05 | 0.05 | 17.50 | > 0.99 |
| ***CF231*** | 0.00 | 0.00 | 0.32 | 0.32 | 17.50 | > 0.99 |
| ***Prevotella (total)*** | 0.07 | 0.04 | 42.24 | 11.63 | 3.50 | **0.01** |
| ***p-2534-18B5*** | 0.00 | 0.00 | 0.95 | 0.95 | 17.50 | > 0.99 |
| ***Cloacibacterium*** | 0.00 | 0.00 | 0.67 | 0.67 | 17.50 | > 0.99 |
| ***YS2*** | 0.00 | 0.00 | 0.15 | 0.15 | 17.50 | > 0.99 |
| ***Elusimicrobiaceae*** | 0.00 | 0.00 | 0.01 | 0.01 | 17.50 | > 0.99 |
| ***Fibrobacter*** | 0.00 | 0.00 | 1.53 | 0.76 | 12.50 | 0.23 |
| ***Lysinibacillus*** | 0.04 | 0.04 | 0.40 | 0.40 | 19.00 | > 0.99 |
| ***Clostridiales*** | 0.00 | 0.00 | 3.92 | 2.27 | 12.50 | 0.23 |
| ***Clostridium*** | 21.88 | 19.11 | 0.88 | 0.88 | 13.50 | 0.32 |
| ***Lachnospiraceae*** | 0.02 | 0.02 | 0.05 | 0.05 | 19.00 | > 0.99 |
| ***Butyrivibrio*** | 0.01 | 0.01 | 1.11 | 0.99 | 18.00 | 0.67 |
| ***Coprococcus*** | 0.00 | 0.00 | 0.07 | 0.07 | 17.50 | > 0.99 |
| ***Pseudobutyrivibrio*** | 0.00 | 0.00 | 1.08 | 1.08 | 17.50 | > 0.99 |
| ***Shuttleworthia*** | 0.00 | 0.00 | 0.51 | 0.51 | 17.50 | > 0.99 |
| ***Peptostreptococcaceae*** | 0.02 | 0.02 | 0.00 | 0.00 | 16.00 | 0.38 |
| ***Ruminococcaceae*** | 0.00 | 0.00 | 1.46 | 1.03 | 12.50 | 0.23 |
| ***Veillonellaceae*** | 0.00 | 0.00 | 1.18 | 1.18 | 17.50 | > 0.99 |
| ***Dialister*** | 0.00 | 0.00 | 0.02 | 0.02 | 17.50 | > 0.99 |
| ***Megasphaera*** | 1.79 | 1.79 | 0.05 | 0.05 | 18.00 | 0.74 |
| ***Selenomonas*** | 0.00 | 0.00 | 0.74 | 0.74 | 17.50 | > 0.99 |
| ***Succiniclasticum*** | 0.00 | 0.00 | 3.01 | 1.51 | 12.50 | 0.23 |
| ***Helcococcus*** | 0.02 | 0.02 | 0.00 | 0.00 | 16.00 | 0.38 |
| ***Fusobacterium*** | 21.71 | 14.81 | 0.00 | 0.00 | 12.00 | 0.13 |
| ***Leptotrichiacea*** | 13.71 | 10.68 | 0.00 | 0.00 | 8.00 | **0.04** |
| ***RF32*** | 0.00 | 0.00 | 0.87 | 0.87 | 17.50 | > 0.99 |
| ***Agrobacterium*** | 0.00 | 0.00 | 0.06 | 0.06 | 17.50 | > 0.99 |
| ***Sphingomonas*** | 0.00 | 0.00 | 2.09 | 2.09 | 17.50 | > 0.99 |
| ***Delftia*** | 1.13 | 0.79 | 0.41 | 0.41 | 11.00 | 0.19 |
| ***Campylobacter*** | 0.02 | 0.02 | 0.00 | 0.00 | 16.00 | 0.38 |
| ***Succinivibrionaceae*** | 0.00 | 0.00 | 7.89 | 4.73 | 12.50 | 0.23 |
| ***Enterobacteriaceae*** | 0.00 | 0.00 | 12.50 | 12.50 | 17.50 | > 0.99 |
| ***Proteus*** | 0.01 | 0.01 | 0.00 | 0.00 | 16.00 | 0.38 |
| ***Pasteurellaceae (total)*** | 8.11 | 5.46 | 0.40 | 0.40 | 6.50 | **0.02** |
| ***Actinobacillus*** | 0.10 | 0.09 | 0.00 | 0.00 | 12.00 | 0.13 |
| ***Pasteurella*** | 0.04 | 0.04 | 0.00 | 0.00 | 16.00 | 0.38 |
| ***Psychrobacter*** | 0.02 | 0.01 | 0.00 | 0.00 | 12.00 | 0.13 |
| ***Stenotrophomonas*** | 0.02 | 0.02 | 0.00 | 0.00 | 16.00 | 0.38 |
| ***Treponema*** | 0.00 | 0.00 | 1.42 | 1.42 | 17.50 | > 0.99 |
| ***Mycoplasma*** | 21.33 | 19.67 | 0.05 | 0.05 | 0.00 | **<0.01** |
