# Supplementary material for: Illumina MiSeq 16S amplicon sequence analysis of bovine respiratory disease associated bacteria in lung and mediastinal lymph node tissue
Source: BMC Vet Res. 2017 May 2;13:118. doi: 10.1186/s12917-017-1035-2 (PMC5414144; doi:10.1186/s12917-017-1035-2)
Supplement: Supplementary file 9 — Description of the results of the PCR and culture tests carried out at the regional veterinary laboratories and the corresponding results of the OTUs identified by 16S rRNA amplicon sequencing. (DOCX 24 kb) [file 12917_2017_1035_MOESM9_ESM.docx]

**Additional file 9.** **Description of the results of the PCR and culture tests carried out at the regional veterinary laboratories and the corresponding results of the OTUs identified by 16S rRNA amplicon sequencing.**

| **Calf ID** | **PCR bacteria** | | **PCR viruses** | **Culture** | **OTU identified (lung)** | **OTU identified (lymph node)** | |
| --- | --- | --- | --- | --- | --- | --- | --- |
| **1** | Negative for *H. somni* | | Negative for Bo-HV1, BRSV, BPIV-3, BCoV | Sterile,  Negative for *Salmonella* | *Leptotrichiaceae, Pasteurella, Mycoplasma, Ureaplasma* | N/A | |
| **2** | Not tested | | Negative for Bo-HV1, BRSV, BPIV-3, BCoV | Not tested | *Leptotrichiaceae, Sphingomonas, Mycoplasma, Lysinibacillus, Lachnospiraceae, Enterobacteriaceae* | N/A | |
| **3** | Positive for *M. bovis* | | Positive for Bo-HV1, Negative for BRSV, BCoV | Positive for *E. coli*.  Negative for *Salmonella* | *Bacteroides, Prevotella, Streptococcus, Helcococcus, Enterobacteriaceae, Pasteurellaceae*, Mycoplasma* | N/A | |
| **4** | Positive for *M. bovis* | | Positive for Bo-HV1, Negative for BRSV, BPIV-3, BCoV | Positive for *E. coli*.  Negative for *Salmonella* | *Mycoplasma* | N/A | |
| **5** | Positive for *H. somni* | | Negative for Bo-HV1, BRSV, BPIV-3, BCoV | Sterile | *Trueperella, Micrococcaceae, Coriobacteriaceae, Bacteroidales, Bacteroides, Porphyromonas, RF16, S24-7, CF231, Prevotella, Endomicrobia, Clostridiales, Christensenellaceae, Lachnospiraceae, Butyrivibrio, Ruminococcaceae, Veillonellaceae, Succiniclasticum, FRN20, Fusobacterium, Leptotrichiaceae, Pirellulaceae, Pasteurellaceae, Actinobacillus, SR1, Treponema, TM7-3, F16, Mycoplasma, Ureaplasma* | N/A | |
| **6** | Not tested | | Positive for BPIV-3, Negative for Bo-HV1, BRSV, BCoV | Positive for *M. haemolytica, Pasteurella* spp.,  Negative for *Salmonella* | *Bacteroides, Pasteurellaceae, Xanthomonadaceae* | N/A | |
| **7** | Positive for *M. bovis* | | Negative for Bo-HV1, BRSV, BPIV-3, BCoV | Positive for *Pseudomonas* spp.  Negative for *Salmonella* | *Porphyromonas, Bacillus, Fusobacterium, Leptotrichiaceae, Pasteurellaceae, Mycoplasma* | *Butyrivibrio, Enterobacteriaceae, Xanthomonadaceae* | |
| **8** | Not tested | | Negative for Bo-HV1, BRSV, BPIV-3, BCoV | Positive for *T. pyogenes*.  Negative for *Salmonella* | *Trueperella, Bacteroides, Clostridium, Leptotrichiaceae, Campylobacter, Psychrobacter, Mycoplasma, Ureaplasma* | *Mycoplasma* | |
| **9** | Positive for *H. somni,* Negative for *M. bovis* | | Positive for Bo-HV1, Negative for BRSV, BPIV-3, BCoV | Positive for *T. pyogenes*.  Negative for *Salmonella* | *Trueperella, Bacteroides, Helcococcus, Fusobacterium, Leptotrichiaceae, Agrobacterium, Enterobacteriaceae, Pasteurellaceae, Actinobacillus, Mycoplasma* | *Trueperella, Bacteroides, Helcococcus, Fusobacterium, Leptotrichiaceae, Campylobacter, Pasteurellaceae, Actinobacillus, Mycoplasma* | |
| **10** | Not tested | | Negative for Bo-HV1, BRSV, BPIV-3, BCoV | Positive for *P. multocida, S. dublin* | *Bacteroides, Porphyromonas, Chitinophagaceae, Clostridium, Fusobacterium, Leptotrichiaceae, Pasteurellaceae, Pasteurella, Mycoplasma, Ureaplasma* | *Trueperella, Bacteroides, Porphyromonas, Clostridium, Fusobacterium, Leptotrichiaceae, Delftia, Proteus, Pasteurellaceae, Psychrobacter, Mycoplasma* | |
| **11** | Negative for *H. somni* | | Negative for Bo-HV1, BRSV, BPIV-3, BCoV | Sterile,  Negative for *Salmonella* | *Fusobacterium, Leptotrichiaceae, Mycoplasma* | *Xanthomonadaceae, Mycoplasma* | |
| **12** | Not tested | | Negative for Bo-HV1, BRSV, BPIV-3, BCoV | Sterile | *Lactobacillus, Enterobacteriaceae, Proteus, Pasteurellaceae, Actinobacillus, Pseudomonas* | *Prevotella, Lactobacillus, Megasphaera, Succiniclasticum, Enterobacteriaceae, Proteus, Pasteurellaceae, Pseudomonas* | |
| **13** | Positive for *M. haemolytica.*  Negative for *H. somni* | | Negative for Bo-HV1, BRSV, BCoV | Positive for *M. haemolytica*,  Negative for *Salmonella* | *Fusobacterium, Delftia, Pasteurellaceae, Actinobacillus* | *Pasteurellaceae* | |
| **14** | Not tested | | Negative for Bo-HV1, BRSV, BPIV-3 | Positive for *P. multocida*,  Negative for *Salmonella* | *Bifidobacterium, Bacteroidales, Prevotella, Lysinibacillus, Lactobacillus, Streptococcus, Lachnospiraceae, Butyrivibrio, Veillonella, Fusobacterium, Pasteurellaceae, Pasteurella, Mycoplasma* | *Bacteroidales, Prevotella, Lactobacillus, Ruminococcaceae, Veillonella,* *Sharpea, Fusobacterium, Pasteurellaceae* | |
| **15** | Not tested | | Positive for Bo-HV1, Negative for BRSV, BPIV-3 | Positive for *E. coli*,  Negative for *Salmonella* | *Trueperella, Coriobacteriaceae, Bacteroides, Porphyromonas, CF231, Prevotella, Lactobacillus, Streptococcus, Clostridiaceae,* *Lachnospiraceae,* *Oribacterium, Filifactor, Ruminococcaceae, Megasphaera, Mogibacteriaceae, Fusobacterium, Leptotrichiaceae, Campylobacter, Enterobacteriaceae, Proteus, Pasteurellaceae, Pasteurella, Pyramidobacter, Mycoplasma* | *Trueperella, Bacteroides, Porphyromonas, CF231, Prevotella, Streptococcus, Clostridiaceae, Clostridium, Filifactor, Ruminococcaceae, Megasphaera, Phascolarctobacterium, Mogibacteriaceae, Sharpea, Fusobacterium, Leptotrichiaceae, Proteus, Pasteurellaceae, Gallibacterium, Xanthomonadaceae, Sphaerochaeta, Pyramidobacter, Mycoplasma* | |
| **16** | Not tested | | Positive for Bo-HV1 and BRSV,  Negative for BPIV-3 | Positive for *Pastuerella* spp.,  Negative for *Salmonella* | *Trueperella, Bacteroides, Porphyromonas, Prevotella, Cloacibacterium, Streptococcus, Helcococcus, Leptotrichiaceae, Pasteurellaceae, Actinobacillus, Mycoplasma, Ureaplasma* | *Bacteroides, Porphyromonas, Megasphaera, Pasteurellaceae, Actinobacillus* | |
| **17** | Not tested | | Negative for BRSV, BPIV-3, BCoV | Sterile,  Negative for *Salmonella* | *Micrococcaceae, Streptococcus, Veillonella, Agrobacterium, Enterobacteriaceae, Pasteurellaceae, Actinobacillus, Moraxella* | *Streptococcus,* *Pasteurellaceae* | |
| **18** | Not tested | | Negative for BRSV, BPIV-3 | Positive for *T. pyogenes*.  Negative for *Salmonella* | *Streptococcus, Leptotrichiaceae, Pasteurellaceae, Pasteurella, Mycoplasma, Ureaplasma* | *Cloacibacterium, Streptococcus, Clostridium, Succiniclasticum, Fusobacterium, Leptotrichiaceae, Tepidimonas, Cupriavidus, Pasteurellaceae, Pasteurella, TM7-3, Mycoplasma* | |
| **19** | Negative for *M. bovis* | Negative for Bo-HV1, BRSV, BPIV-3, BCoV | | Positive for  *E. coli* | *Bifidobacterium, Bacteroides, Prevotella, Streptococcus, Clostridiales, Phascolarctobacterium, Leptotrichiaceae, Comamonas, Enterobacteriaceae, Pasteurellaceae, Gallibacterium, Psychrobacter, Mycoplasma* | *Bifidobacterium, Bacteroides, Prevotella, Phascolarctobacterium, Sharpea, Comamonas, Delftia, Enterobacteriaceae, Gallibacterium, Psychrobacter* |  |
| **21** | Positive for *M. bovis* | Negative for Bo-HV1, BRSV, BPIV-3, BCoV | | Positive for  *H. somni* | *Cloacibacterium, Leptotrichiaceae, Agrobacterium, Neisseriaceae, Campylobacter, Enterobacteriaceae, Pasteurellaceae, Mycoplasma, Ureaplasma* | *Trueperella, Bacteroidales, Bacteroides Porphyromonas, RF16, S24-7, Prevotella, Endomicrobia, Fibrobacter, Lactobacillus, Streptococcus,* *Clostridiales, Clostridium, Lachnospiraceae, Butyrivibrio, Oribacterium, Ruminococcaceae, Ruminococcus, Anaerovibrio, Selenomonas, Succiniclasticum, Fusobacterium, Leptotrichiaceae, Succinivibrionaceae, Enterobacteriaceae, Proteus, Pasteurellaceae, Actinobacillus, Pasteurella, Xanthomonadaceae, Treponema, F16, Mycoplasma, RFP12* |  |
| **22** | Negative for *M. bovis* | Negative for Bo-HV1, BRSV, BPIV-3, BCoV | | Positive for  *P. multocida,*  *H. somni* | *Coriobacteriaceae, Lysinibacillus, Leptotrichiaceae, Pasteurellaceae, Pasteurella, Mycoplasma* | *Streptophyta, Lysinibacillus, Leptotrichiaceae, Pasteurellaceae, Xanthomonadaceae, Mycoplasma* |  |
| **23** | Negative for *M. bovis* | Negative for Bo-HV1, BRSV, BPIV-3, BCoV | | Positive for  *H. somni* | *Trueperella, Paraprevotellaceae, Prevotella, Streptococcus, Fusobacterium, Leptotrichiaceae, Succinivibrionaceae, Enterobacteriaceae, Pasteurellaceae, Mycoplasma* | *Prevotella, Facklamia, Succiniclasticum, Leptotrichiaceae, ZB2, Cupriavidus, Pasteurellaceae* |  |
| **24** | Not tested | Negative for Bo-HV1, BRSV, BPIV-3, BCoV | | Positive for *Streptococcus* spp.,  Negative for *Salmonella* | *Clostridiaceae, Clostridium, Peptostreptococcaceae, Fusobacterium, Leptotrichiaceae, Pasteurellaceae, Mycoplasma* | *Clostridiaceae, Clostridium, Peptostreptococcaceae, Fusobacterium, Agrobacterium* |  |
| **25** | Not tested | Negative for Bo-HV1, BRSV, BPIV-3, BCoV | | Positive for *Proteus* spp. Negative for *Salmonella* | *Actinomyces, Trueperella, Prevotella, Granulicatella, Streptococcus, Clostridium, Catonella, Veillonella, Fusobacterium, Leptotrichiaceae, Proteus, Pasteurellaceae, Actinobacillus, Gallibacterium, Pasteurella, TM7-3* | *Trueperella, Arthrobacter, Coriobacteriaceae, Bacteroidales, Bacteroides, RF16, Paraprevotellaceae, Prevotella, Elusimicrobiaceae, Fibrobacter, Streptococcus, Clostridiales, Christensenellaceae, Clostridiaceae, Lachnospiraceae, Butyrivibrio, Coprococcus, Ruminococcaceae, Ruminococcus, Veillonellaceae, Megasphaera, Succiniclasticum, RFN20, Sharpea, Fusobacterium, Leptotrichiaceae, Pirellulaceae, Succinivibrionaceae, Proteus, Pasteurellaceae, Actinobacillus, Pasteurella, SR1, Treponema, F16, Mycoplasma, Ureaplasma* |  |
| **26** | Negative for *H. somni* | Negative for Bo-HV1, BRSV, BPIV-3, BCoV | | Positive for *E.coli*.  Negative for *Salmonella* | *Coriobacteriaceae, Bacteroides, Prevotella, Streptococcus, Clostridiales, Shuttleworthia, Helcococcus, Fusobacterium, Enterobacteriaceae, Pasteurellaceae, Actinobacillus, Pasteurella, SR1, Mycoplasma* | *Clostridium, Enterobacteriaceae, Proteus, Pasteurellaceae, Pasteurella* |  |
| **27** | Not tested | Positive for Bo-HV1, Negative for BRSV, BPIV-3, BCoV | | Positive for *E. coli.*  Negative for *Salmonella* | *Arthrobacter, Coriobacteriaceae, Bacteroides, S24-7, Prevotella,* *Clostridiales, Lachnospiraceae, Acidaminococcus, Phascolarctobacterium, Succiniclasticum, Fusobacterium, Anaplasma, Proteus, Pasteurellaceae, Gallibacterium, Sphaerochaeta, Pyramidobacter* | *Coriobacteriaceae, Prevotella, Streptococcus, Lachnospiraceae, Butyrivibrio,* *Ruminococcaceae, Fusobacterium, Leptotrichiaceae, Anaplasma, Pasteurellaceae, Gallibacterium* |  |
| **28** | Not tested | Positive for Bo-HV1, Negative for BRSV, BPIV-3, BCoV | | Positive for Proteus spp., Negative for *Salmonella* | *Actinomyces, Rothia, Porphyromonas, Prevotella, Streptophyta, Gemellaceae, Granulicatella, Streptococcus, Clostridium, Catonella, Veillonella, Helcococcus, Fusobacterium, Leptotrichiaceae, Leptotrichia, Neisseria, Campylobacter, Proteus, Pasteurellaceae, TM7-3, Mycoplasma, Ureaplasma* | *Bacteroides, Streptococcus, Agrobacterium, Novosphingobium, Pasteurellaceae* |  |
| **29** | Not tested | Negative for Bo-HV1, BRSV, BPIV-3, BCoV | | Not tested | *Arthrobacter, Coriobacteriaceae, Bacteroidales, BS11, Bacteroides, CF231, Prevotella, Clostridiales, Christensenellaceae, Clostridiaceae, Clostridium, Pseudobutyrivibrio, Ruminococcaceae, Veillonellaceae, Succiniclasticum, RFN20, Fusobacterium, Victivallaceae, Campylobacter, Enterobacteriaceae, Pasteurellaceae, Acinetobacter, Mycoplasma* | *Arthrobacter, Bacteroides, Prevotella, Streptococcus, Clostridium, Oscillospira, Victivallaceae, Campylobacter, Enterobacteriaceae, Acinetobacter, Pseudomonas* |  |
| **30** | Positive for *M. bovis*.  Negative for *H. somni* | Negative for Bo-HV1, BRSV, BCoV | | Sterile,  Negative for *Salmonella* | *Microbacteriaceae, Coriobacteriaceae, Bacteroidales, Prevotella, Fibrobacter,* *Bacillus, Lactobacillus, Clostridiales, Clostridium, Ruminococcaceae, Succiniclasticum,* *Sharpea,* *Delftia* | *Prevotella, Bacillus, Lactobacillus* |  |
| **31** | Positive for *M. bovis.*  Negative for *H. somni* | Negative for Bo-HV1, BRSV, BPIV-3, BCoV | | Sterile,  Negative for *Salmonella* | *Microbacteriaceae, Arthrobacter, Clostridium, Sharpea, Leptotrichiacea, Mycoplasma* | *Clostridium* |  |
| **32** | Negative for *M. bovis* | Negative for Bo-HV1, BRSV, BPIV-3, BCoV | | Sterile | *Bacteroides, Prevotella, Lactobacillus, Butyrivibrio, Selenomonas, Fusobacterium, Leptotrichiaceae, Agrobacterium, Comamonas, Neisseriaceae, Pasteurellaceae, Mycoplasma, WPS-2* | *Chitinophagaceae, Endomicrobia, Lactobacillus, Streptococcus, Fusobacterium, Leptotrichiaceae, Pasteurellaceae, Actinobacillus, Treponema* |  |
| **33** | Not tested | Negative for Bo-HV1, BRSV, BPIV-3, BCoV | | Positive for *P. multocida* | *Trueperella, Microbacteriaceae, Bacteroides, Porphyromonas, Prevotella, Clostridium, Fusobacterium, Leptotrichiaceae, Pasteurellaceae, Pasteurella, Acinetobacter, Mycoplasma* | *Microbacteriaceae, Bifidobacterium, Coriobacteriaceae, Prevotella, Lysinibacillus, Clostridium, Lachnospiraceae, Butyrivibrio,* *Peptostreptococcaceae, Delftia, Pasteurellaceae, Pasteurella, Actinobacillus, Psychrobacter, Mycoplasma* |  |
| **34** | Not tested | Negative for Bo-HV1, BRSV, BPIV-3, BCoV | | Positive for *P. multocida, T. pyogenes* | *Trueperella, Microbacteriaceae, Bacteroides, Porphyromonas, Prevotella, Bacillus, Clostridium, Helcococcus, Fusobacterium, Leptotrichiaceae, Delftia, Enterobacteriaceae, Pasteurellaceae, Actinobacillus, Pasteurella, Mycoplasma, Ureaplasma* | *Trueperella, Arthrobacter, Bacteroidales, Bacteroides, Porphyromonas, Prevotella, Clostridium, Butyrivibrio, Coprococcus, Ruminococcaceae, Succiniclasticum, Helcococcus, Fusobacterium, Leptotrichiaceae, Agrobacterium, Rickettsiales, Pasteurellaceae, Xanthomonadaceae, Stenotrophomonas, Mycoplasma* |  |
| **35** | Negative for *M. bovis* | Negative for Bo-HV1, BRSV, BPIV-3, BCoV | | Sterile | *Microbacteriaceae, Sphingomonas, Delftia, Pasteurellaceae, Mycoplasma* | *Clostridium,* *Cupriavidus, Pasteurellaceae, Xanthomonadaceae* |  |
| **36** | Negative for *M. haemolytica*, *H. somni* | Negative for Bo-HV1, BRSV, BPIV-3, BCoV | | Sterile,  Negative for *Salmonella* | *Coriobacteriaceae, Prevotella, Chlamydia, Clostridium, Butyrivibrio, Acidaminococcus, Selenomonas, Sharpea, Agrobacterium, Anaplasma, Pasteurellaceae, Mycoplasma, Ureaplasma* | *Bifidobacterium, Coriobacteriaceae, S24-7, Prevotella, Lactobacillus, Streptococcus, Clostridiales, Clostridium, Lachnospiraceae, Butyrivibrio, Ruminococcaceae, Veillonellaceae, Acidaminococcus, Anaerovibrio, Megasphaera, Phascolarctobacterium, Selenomonas, Succiniclasticum, Mogibacteriaceae, Sharpea, Victivallaceae, Anaplasma, Sphaerochaeta, Pyramidobacter* |  |
| **37** | Positive for *M. haemolytica,*  Negative for *M. bovis*, *H. somni* | Negative for Bo-HV1, BRSV, BPIV-3, BCoV | | Positive for *M. haemolytica*.  Negative for *Salmonella* | *Lactobacillus, Clostridium,* *Veillonella, Fusobacterium, Delftia, Pasteurellaceae, Mycoplasma* | *Coriobacteriaceae,* *Bacteroidales, Bacteroides, S24-7, Paraprevotellaceae, Prevotella, Lactobacillus, Streptococcus, Clostridium, Lachnospiraceae, Butyrivibrio, Acidaminococcus, Megasphaera,* *Veillonella, Fusobacterium, Pasteurellaceae, Xanthomonadaceae, F16, Mycoplasma* |  |
| **38** | Positive for *M. haemolytica, M. bovis, H. somni, P. multocida* | Negative for Bo-HV1, BRSV, BPIV-3, BCoV | | Positive for *T. pyogenes*.  Negative for *Salmonella* | *Trueperella, Bacteroides, Porphyromonas, CF231, Prevotella, Chlamydia, Streptococcus, Helcococcus, Fusobacterium, Leptotrichiaceae, Campylobacter, Pasteurellaceae, Pasteurella, Ureaplasma* | *Trueperella, Bacteroides, Porphyromonas, CF231, Prevotella, Chryseobacterium, Cloacibacterium, Bacillus, Fusobacterium, Leptotrichiaceae, Agrobacterium, Campylobacter, Pasteurellaceae, Mycoplasma* |  |

*E. coli = Escherichia coli,* *H. somni = Histophilus somni, M. bovis = Mycoplasma bovis, M. haemolytica =* [*Mannheimia haemolytica*](https://www.google.ie/search?biw=1345&bih=628&q=Mannheimia+haemolytica&spell=1&sa=X&ved=0ahUKEwjI3vmWuIHKAhXIwA4KHWqzBGcQvwUIGCgA)*, P. multocida = Pasteurella multocida, S. dublin = Salmonella dublin, T. pyrogenes = Trueperella pyrogenes,* BRSV = bovine respiratory syncytial virus, BPIV-3 = parainfluenza 3 virus, Bo-HV1 = bovine herpesvirus 1, BCoV = bovine coronavirus, OTU = operational taxonomic unit.

N/A = not applicable as tissue was not collected.
