# Supplementary material for: Illumina MiSeq 16S amplicon sequence analysis of bovine respiratory disease associated bacteria in lung and mediastinal lymph node tissue
Source: BMC Vet Res. 2017 May 2;13:118. doi: 10.1186/s12917-017-1035-2 (PMC5414144; doi:10.1186/s12917-017-1035-2)
Supplement: Supplementary file 10 — Assembled bacterial contig sequences. (DOCX 30 kb) [file 12917_2017_1035_MOESM10_ESM.docx]

**Additional file 10.** **Assembled bacterial contig sequences.**

| **Contig I.D.** | **Contig sequence** |
| --- | --- |
| **1** | CTGTAAGAGGAATATAGTTGGAAAGCTAAACCAAAGAAGGTGATAGTCCTGTAGATTGTAGAGTATATATATATCTGGTATAAACCCCGAGTAGCATCAAGCACGAGGAATTTGGTGTGAATCAGTGAGGACCATATCTCATAAGGCTAAATACTTTTACTAACCGATAGTGAAGAGTACCGTGAGGGAAAGGTGAAAAGAACCCTGAGTAAGGGAGTGAAATAGAATTTGAAACCGTACGCTTACAAGCGGTAGGAGCAGGGTAACCTGTGACTGCGTGGATTTTGGTTAATCATCCTGCGAGTTATGATATATGGCAAGGTTAAGGAAAGCGGAGCCGAAGGGAAACCAAGTCTTAAGAGGGCGAAAGTCGTATGTCATAGACGCGAAACCTAGTGATCTAGGCCTGTCCAAGTTGAAGCTAAGGTAAGACTTAGTGGAGGACTGAACTCACCGCCGTTGAAATGATGGGAGATGAGATAGGTTTAGGGGTGAAAAGCCAATCGAACTAGGAGATAGCTCGTTCTCTCCGAAATGCATTTAGGTGCAGCCTTAATGGAAGATATGTGGGGGTAGAGCACTGTATGGCCTAGGGGGCGTATAGCTTACTGAAGTCAAGCAAACTACGAATACCATATATTAATAATTAGGAGTGAGTCTATGGATGACAAGGTCCATGGACGAGAGGGAAAAAGCCCAGAACAACAGCTAAGGTCCCGAATTATGTCTAAGTGGGAAAGGAGGTGGATATTCAAAGACAACCAGGAGGTTGGCTTAGAAGCAGCCAAGCCTTAAAAGAGTGCGTAACAGCTCACTGGTCGAGAGTATCTGCGCCGAAGATTTAACGGGGCTAAGACATAAACCGAAGCTTTGTAACATAATGTAAGTTATGTTGGTAGGAGAGCGTTCTGTAAGCCGTAGAAGGAGGATTGAAAGAGAATCTGGAGGTATCAGAAGTGAGAATGCAGGAATGAGTAGCGAGAAAGAGGGTGAGAATCCCTCTGACCGGAAGTCCAAGGATTCCAGGGGAAGGTTTGTCCGCCCTGGGGGAGTCGGGACCTAAGGATAAACAGAAATGTGAAGCCGAATGGAAAATAGGTAAATATTCCTATACCACTTAATTAACGATTGAAGGAATGGAGTGACGCAGGAAGGTATGTAAGATGACGGACGGAAGAGTCATTTTAAGGAAGAAGCATGAGTATATAGGCAAATCCGTATACTTAGATGTGAGAACTGAAGAGGAAGCATATGTAAAGTATGTAAGTTACAAATCCTATACTGCCGAGAAAAACTTCTACCGAGGAGATTAAGTGCCCGTACCGCAAACCGACACAGGTGGACAGGGTGAGAAACCTAAGGTCGACAGGCTAACTCTAGCTAAGGAACTCTGCAAAATAGCCCCGTAACTTCGGGAGAAGGGGTACCTATGAAAGTGAGTAAAGAGACATTACGAGCAAGAGTAGGTCGCAGTGAAGAGTCCCAAGCAACTGTTTACCAAAAACACAGGTCTATGCTAAGCCGGAAGGCGACGTATATGGGCTGACACCTGCCCAGTGCTGGAAGGTTAAGAGGAGGATTGAGAGATTCGAATTGAAGCCCCAGTGAACGGCGGCCGTAACTATAACGGTCCTAAGGTAGCGAAATTCCTTGTCAGGTAAGTTCTGACCTGCACGAATGGTGCAATGATTTGGGAGCTGTCTTGGCTGGAGGCCTGGTGAAGTTGTAATAGCGGTGAAGATACCGCTTACCTGCAGTAGGACGGAAAGACCCCGTGGAGCTTTACTGTAGTTTGGCATTGGGTTTTGGCAATGTGTGTATAGGATAGTTGGGAGACGAAGAGATAAGTACGCAAGTATTTAAGGAGTCGGTGTTGGAATACCAACCATATATTGTTGAAATTCTAATCAATAAAATGAGACAGTGCTAGATGGGCAGTTTGACTGGGGCGGTCACCTCCAAAAGAGTAACGGAGGTGTTCAAAGGTTCCCTCAGGTTGGATGGAAATCAACCAGAGAGTGTAAAGGCACAAGGGAGCTTGACTGCGAGACTGACGGGTCGAGCAGGTACGAAAGTAGGACTTAGTGATCCGGTGGTACTGAATGGAAAGGCCATCGCTCAACGGATAAAAGCTACCCCGGGGATAACAGGCTGATACTTCCCAAGAGTCCATATCGACGGAAGTGTTTGGCACCTCGATGTCGGCTCGTCTCATCCTGGGGCTGGAGAAGGTCCCAAGGGTTGGGCTGTTCGCCCATTAAAGAGGCACGCGAGCTGGGTTCAGAACGTCGTGAGACAGTTCGGTCCCTATCCACTGCAGGCGAAAGAGTATTGAGAAGAACTGTCCTTAGTACGAGAGGACCGGGATGGACGAATCACTGATGTACCAGTTGTATCGCCAGATGCATAGCTGGGTAGTCACATTCGGGAAGGATAATCGCTGAAAGCATCTAAGTGAGAAGCCAGCTTCAAGATAAGTACTCTGTTAGTACCCACCGAGACTAGGTGGTAGATAGGCTAGGGGTGTAAGTGTAGTAATACATTAAGCTGACTAGTACTAATGAGACGAAAGCTTAAGAAACTAATGAAGGAACTTTTTACCTAACTATTTATTTCTAATTGTCTTATAGACAAGAGTTTGGTGACA |
| **2** | GAATTATTGGGCTTAAAGGGCATCTAGGCGGCATAACAAGTTGAAGGTGAAAAACTTAGGCTCAACCAAAGTCTTGCCTACAAAACTGTAATGCTAGAGTACTGGAAAGGTGGGTGGAACTACACGAGTAGAGGTGAAATTCGTAGATATGTGTAGGAATGCCGATGATGAAGATAACTCACTGGACAGCTACTGACGCTGAAGTGCGAAAGCTAGGGGAGCAAACAGGATTAGATACCCTGGTAGTCCTAGCTGTAAACGATGATTACTGGGTGTGGGGATGAGAAGTCTCTGTGCCGAAGCAAAAGCGATAAGTAATCCGCCTGGGGAGTACGTTCGCAAGAATGAAACTCAAAGGAATTGACGGGGGCCCGCACAAGTGGTGGAGCATGTGGTTTAATTCGACGCAACGCGAGGAACCTTACCAGATCTTGACATCCTCGGAAAGTTATAGAGATATGACAGTGCCTTAGGGAACCGAGAGACAGGTGGTGCATGGCTGTCGACAGCTCGTGTTGTGAGATGTTGGGTTAAGTCCCGCAACGAGCGAAACCCCTATCATTAGTTACCATCATTAAGTTGGGGACTCTAATGAAACTGCCTGCGAAGAGCAGGAGGAAGGTGGGGATGACGTCAAGTCATCATGCCCCTTATGATCTGGGCTACACACGTGCTACAATGGGTAGTACAAAGAGAGGCGAAATGGCGACATGGAGCAAAACTAGAAAGCTACTCTAAGTTCGGATTGAAGTCTGCAACTCGACTTCATGAAGTTGGAATCACTAGTAATCGCAAATCAGCAATGTTGCGGTGAATACGTTCTCGGGCCTTGTACACACCGCCCGTCACACCACGAGAGTTGTTTGCACCTGAAATTATTGGTCTAACCGTAAGGGGGAAGATAATGAAGGTGTGAATAGTGATTGGGGTGAAGTCGTAACAAGGTATCCGTACCGGAAGGTGCGGATGGATCACCTCCTTTCTAAGGAGAAAGAAGATAACATTTGTTTGCTTTTATTCTTTTAATTTATGGGCGTGTAGCTCAGGTGGTTAGAGCACTGTGCTGATAACGCAGGGGTCGATGGTTCGAGTCCATTCATGCCCACCATAAAAAACTTAAGTATGGGGATATAGCTCAGCTGGGAGAGCGCCGCACTTGCACTGCGGAGGTCAGCAGTTCGATCCTGCTTATCTCCACCAAGAAAAAGACATAGGACAATGAGAAATGAATAGTAGGTAAAAAGAAAATAACAACATTCACAAAAGAAGAAGAAGAGTTAGAATGATAAGCTAAGAAAGGGCGTACGGAGGATGCCTAGGTAGTAAAAGCCGAAGAAGGACGTGATAAGCTGCGAAAAGCTAGGTGTAGTTGCAAA |
| **3** | CCCTAAGGCGAGGCTGAAAAGCGTAGTTGATGGGAAACGGGTTAATATTCCCGTACTTTGATAAACTGCGATGTGGGGACGGAGAAGGTTAGGTTATCGACCTGTTGGATGGTCGTTTAAGCCTGTAGGTGGGAAGATTAGGCAAATCCGGTCTTCTATTAAACGCTGAGGGGTGAAGAGGAGTTTCTAAGGAGACGAAGTAACTGATACCACGCTTCCAGGAAAAGCCACTAAGCGAAAGGTTTATTGGAACCGTACTGAAAACCGACACAGGTGGTCAGGTAGAGAATACTCAGGCGCTTGAGAGAACTCGGGTGAAGGAACTAGGCAAAATGGCACCGTAACTTCGGGAGAAGGTGCGCTTACGGTAATTGTAGCTCTTTACGGGTGAAGGTGAAGTAAGTCGAAGATACCAGCTGGCTGCAACTGTTTATTAAAAACACAGCACTCTGCGAACACGAAAGTGGAAGTATAGGGTGTGATGCCTGCCCGGTGCTGGAAGGTTAATTGATGTTGTAATCGAAAGAGAAGCAGCTGATCGAAGCCCCAGTAAACGGCGGCCGTAACTATAACGGTCCTAAGGTAGCGAAATTCCTTGTCGGGTAAGTTCCGACCTGCACGAATGGCATAATGATGGCCAGGCTGTCTCCACCCGAGGCTCAGTGAAATTGAAATCGCCGTGAAGATGCGGTGTACCCGCGGCTAGACGGAAAGACCCCGTGAACCTTTACTATAGCTTGACAC |
